# Sustained effectiveness and cost-effectiveness of the Healthy Activity Programme, a brief psychological treatment for depression delivered by lay counsellors in primary care: 12-month follow-up of a randomised controlled trial

**DOI:** 10.1371/journal.pmed.1002385

**Published:** 2017-09-12

**Authors:** Benedict Weobong, Helen A. Weiss, David McDaid, Daisy R. Singla, Steven D. Hollon, Abhijit Nadkarni, A-La Park, Bhargav Bhat, Basavraj Katti, Arpita Anand, Sona Dimidjian, Ricardo Araya, Michael King, Lakshmi Vijayakumar, G. Terence Wilson, Richard Velleman, Betty R. Kirkwood, Christopher G. Fairburn, Vikram Patel

**Affiliations:** 1 Centre for Global Mental Health, Faculty of Epidemiology and Population Health, London School of Hygiene & Tropical Medicine, London, United Kingdom; 2 MRC Tropical Epidemiology Group, Faculty of Epidemiology and Population Health, London School of Hygiene & Tropical Medicine, London, United Kingdom; 3 Personal Social Services Research Unit, London School of Economics and Political Science, London, United Kingdom; 4 Department of Psychiatry, Sinai Health Network, University of Toronto, Toronto, Ontario, Canada; 5 Department of Psychology, Vanderbilt University, Nashville, Tennessee, United States of America; 6 Sangath, Socorro, Goa, India; 7 Department of Psychology and Neuroscience, University of Colorado, Boulder, Colorado, United States of America; 8 Institute of Psychiatry, Psychology, and Neurosciences, King’s College Hospital, London, United Kingdom; 9 Division of Psychiatry, Faculty of Brain Sciences, University College London, London, United Kingdom; 10 SNEHA, Voluntary Health Services, University of Melbourne, Melbourne, Victoria, Australia; 11 Department of Psychology, School of Arts and Sciences, Rutgers University, New Brunswick, New Jersey, United States of America; 12 Department of Psychology, University of Bath, Bath, United Kingdom; 13 Department of Psychiatry, University of Oxford, Oxford, United Kingdom; 14 Department of Global Health and Social Medicine, Harvard Medical School, Boston, Massachusetts, United States of America; Massachusetts General Hospital, UNITED STATES

## Abstract

**Background:**

The Healthy Activity Programme (HAP), a brief behavioural intervention delivered by lay counsellors, enhanced remission over 3 months among primary care attendees with depression in peri-urban and rural settings in India. We evaluated the sustainability of the effects after treatment termination, the cost-effectiveness of HAP over 12 months, and the effects of the hypothesized mediator of activation on clinical outcomes.

**Methods and findings:**

Primary care attendees aged 18–65 years screened with moderately severe to severe depression on the Patient Health Questionnaire 9 (PHQ-9) were randomised to either HAP plus enhanced usual care (EUC) (*n =* 247) or EUC alone (*n =* 248), of whom 95% completed assessments at 3 months, and 91% at 12 months. Primary outcomes were severity on the Beck Depression Inventory–II (BDI-II) and remission on the PHQ-9. HAP participants maintained the gains they showed at the end of treatment through the 12-month follow-up (difference in mean BDI-II score between 3 and 12 months = −0.34; 95% CI −2.37, 1.69; *p =* 0.74), with lower symptom severity scores than participants who received EUC alone (adjusted mean difference in BDI-II score = −4.45; 95% CI −7.26, −1.63; *p =* 0.002) and higher rates of remission (adjusted prevalence ratio [aPR] = 1.36; 95% CI 1.15, 1.61; *p <* 0.009). They also fared better on most secondary outcomes, including recovery (aPR = 1.98; 95% CI 1.29, 3.03; *p =* 0.002), any response over time (aPR = 1.45; 95% CI 1.27, 1.66; *p <* 0.001), higher likelihood of reporting a minimal clinically important difference (aPR = 1.42; 95% CI 1.17, 1.71; *p <* 0.001), and lower likelihood of reporting suicidal behaviour (aPR = 0.71; 95% CI 0.51, 1.01; *p =* 0.06). HAP plus EUC also had a marginal effect on WHO Disability Assessment Schedule score at 12 months (aPR = −1.58; 95% CI −3.33, 0.17; *p =* 0.08); other outcomes (days unable to work, intimate partner violence toward females) did not statistically significantly differ between the two arms. Economic analyses indicated that HAP plus EUC was dominant over EUC alone, with lower costs and better outcomes; uncertainty analysis showed that from this health system perspective there was a 95% chance of HAP being cost-effective, given a willingness to pay threshold of Intl$16,060—equivalent to GDP per capita in Goa—per quality-adjusted life year gained. Patient-reported behavioural activation level at 3 months mediated the effect of the HAP intervention on the 12-month depression score (β = −2.62; 95% CI −3.28, −1.97; *p <* 0.001). Serious adverse events were infrequent, and prevalence was similar by arm. We were unable to assess possible episodes of remission and relapse that may have occurred between our outcome assessment time points of 3 and 12 months after randomisation. We did not account for or evaluate the effect of mediators other than behavioural activation.

**Conclusions:**

HAP’s superiority over EUC at the end of treatment was largely stable over time and was mediated by patient activation. HAP provides better outcomes at lower costs from a perspective covering publicly funded healthcare services and productivity impacts on patients and their families.

**Trial registration:**

ISRCTN registry ISRCTN95149997

## Abstract 2

In a randomized controlled trial, Vikram Patel and colleagues examine the 12 month effectiveness and cost-effectiveness of a brief psychological treatment for depression, delivered by lay counsellors in primary care.

## Author summary

**Why was this study done?:**

- Depression is the leading mental health contributor to the global burden of disease.
- Access to effective treatments is low globally, but especially so in low- and middle-income countries like India, where a recent national survey reported a treatment gap of 85%.
- The Healthy Activity Programme (HAP) is a brief psychological treatment based on the principles of behavioural activation and delivered by non-specialist providers; we have earlier reported the effectiveness of this intervention in reducing depressive symptoms and promoting remission at the end of treatment.
- The aim of the present study was to evaluate the sustained effectiveness and the cost-effectiveness of HAP over 12 months and to assess whether behavioural activation reported by patients at 3 months mediated the effects of the intervention on depression at 12 months.

**What did the researchers do and find?:**

- We implemented a randomised controlled trial in which 493 adult primary health-care attendees with moderately severe or severe depression were assigned to either the HAP treatment plus enhanced usual care (EUC) (*n =* 245) or EUC alone (*n =* 248); those assigned to HAP received treatment over 2 to 3 months.
- HAP participants maintained the gains they showed at the end of treatment through the 12-month period, with lower symptom severity scores than participants who received EUC alone and higher rates of remission; these effects were partly mediated by increased levels of behavioural activation reported at 3 months.
- HAP was highly likely to be cost-effective, and could even save money if productivity costs were taken into account.

**What do these findings mean?:**

- HAP is associated with sustained effects on depression outcomes over a 12-month period and represents good value for money.
- HAP is ideally suited for scaling up to reduce the treatment gap for depression.

## Introduction

Depression is a major contributor to the global burden of disease [1], and its treatment is a priority in the global health agenda. Despite the well-documented health and economic consequences of depression [2,3], investments in mental health are inadequate, resulting in a large treatment gap [3]. Access to treatment remains a challenge, particularly in low- and middle-income countries (LMICs). The recent National Mental Health Survey in India reported a treatment gap of 85% for major depression [4]. Psychological treatments (PTs) are recommended as first-line interventions [5], not only because they are as efficacious as pharmacological treatments, but also because they produce sustained effects after treatment termination [6]. However, there are questions about the generalisability of PTs in LMICs, where the lack of trained professionals, variations in explanatory models, and lower literacy may present structural barriers to PT [7,8]. Some of these barriers could be overcome by the innovative use of task-sharing, and there is growing evidence for the acceptability and effectiveness of contextually sensitive PTs delivered by appropriately trained and supervised lay health workers in primary care and community settings [9–11]; however, there are very few trials that have reported on the sustained effects, cost-effectiveness, or mediation of the effects of these treatments.

The PRogram for Effective Mental health Interventions in Under-resourced health systeMs (PREMIUM) was designed to (1) implement a methodology for the development of scalable PTs that are culturally appropriate, affordable, and feasible for delivery by non-specialist health workers and (2) evaluate the effectiveness and cost-effectiveness of PTs for the 2 leading mental health causes of the burden of disease, the Counselling for Alcohol Problems (CAP) programme for harmful drinking [12] and the Healthy Activity Programme (HAP) for moderately severe to severe depression [13,14]. The HAP treatment is adapted from behavioural activation, a treatment which has a strong theoretical and empirical evidence base across diverse contexts and patient populations [15]. The stance of behavioural activation is particularly attractive as it focuses on the link between activities and mood, whilst emphasising increased activation and engagement, problem solving skills, and enhanced social support. A core feature of PREMIUM was the delivery of both treatments by the same lay counsellors in routine primary care settings, as they would be used in actual clinical practice. Usual care in primary care for depression in India is, in effect, no care at all. This was confirmed in the study setting during the pilot study. This is primarily because most cases are not diagnosed, and, amongst individuals who are diagnosed, most receive neither antidepressants nor PT.

Previously, we reported the favourable results of the impact of 6–8 sessions of HAP on mental health and secondary outcomes at the primary 3-month post-enrolment endpoint [16]. The key findings were that HAP produced significantly lower symptom severity (adjusted mean difference [AMD] in Beck Depression Inventory–II [BDI-II] score = −7.57; 95% CI −10.27, −4.86) and higher remission rate (adjusted prevalence ratio [aPR] = 1.61; 95% CI 1.34, 1.93). HAP also showed superior results on the secondary outcomes of disability, days out of work, and intimate partner physical violence in women. The incremental cost of HAP per quality-adjusted life year (QALY) gained was Intl$9,333 (95% CI Intl$3,862, Intl$28,169), with an 87% chance of being cost-effective from a health system perspective in the study setting.

The question now becomes whether these effects were sustained following the end of treatment—in a disorder that is highly prone to relapse and recurrence—given HAP’s relatively brief duration, minimal dosage, and delivery by non-specialised workers (most brief PTs, particularly behavioural-activation-based treatments in high-income countries, typically involve at least twice this number of sessions, delivered by highly trained professionals). In addition, a meaningful sustained effect should be accompanied by a patient-defined clinically important improvement in symptoms, as well as evidence that the mediating factor targeted by the PT accounted for the PT’s effects. In this paper, we address 3 novel questions: the stability of HAP’s effects on depression and other outcomes at 12 months post-enrolment, the mediation of the clinical outcomes by patient activation assessed at 3 months, and the cost-effectiveness of the intervention over 12 months.

## Methods

The methods are described in detail in the protocol (S1 Protocol). The trial was conducted in accordance with the protocol (ISRCTN95149997) [17], which was approved by the trial steering committee. Approval for the conduct of the trial was obtained from the institutional review boards of the London School of Hygiene & Tropical Medicine, Sangath (the implementing institution in India), and the Indian Council of Medical Research. Written (or witnessed, if the participant was illiterate) informed consent was mandatory for enrolment. This study is reported as per CONSORT guidelines (S1 CONSORT Checklist).

### Study design and participants

This was a parallel-arm, individually randomised controlled trial in 10 primary health centres in Goa, a state on the west coast of India. Participants were adult primary health centre (PHC) patients aged 18–65 years with a probable diagnosis of moderately severe to severe depression ascertained with a Patient Health Questionnaire 9 (PHQ-9) score > 14, a cut-point previously validated in the study setting, and who gave informed consent. Pregnant women, patients presenting with severe medical conditions requiring urgent medical attention, and those with hearing/speech difficulties were excluded. Participants were interviewed to collect data on socio-demographic factors and potential moderators of treatment outcome: sex, illness severity, duration of the illness, and expectations for treatment [18]. Sequential numbered opaque envelopes were used to randomise consenting participants in a 1:1 allocation scheme [19]. Enrolment was conducted between 28 October 2013 and 29 July 2015, and the final 12-month assessment was completed on 30 August 2016.

### Sample size estimation

Our sample size estimations for the 3-month primary outcomes assumed an intra-cluster correlation between PHCs of 0.04, with 1 counsellor per PHC at any one time, loss to follow-up of 15% over 3 months, and a 1:1 allocation ratio. Based on these assumptions, we aimed to recruit 500 participants (425 in our analysis sample) to detect the hypothesized effects: (1) a standardised mean difference (effect size [ES]) of 0.42 for the primary continuous outcome of depression severity, with 90% power, and (2) a proportion recovered of 65% in the HAP plus enhanced usual care (EUC) arm compared with 44% in the EUC arm, with 92% power. The high follow-up rate (attrition rate of 9%) at 12 months means that we have 90% power to detect these ESs at 12 months.

### Interventions

#### Enhanced usual care

EUC comprised routine consultation with the PHC physician, enhanced by providing the PHQ-9 screening results to both the PHC physician and the patient, and providing copies of a contextualised version of the WHO Mental Health Gap Action Programme (mhGAP) guidelines to the PHC physician that included information on when and where to refer for psychiatric care [20]. EUC was available to all trial participants.

#### Healthy activity programme

HAP is a contextually adapted brief PT based on behavioural activation [13] that focuses on increasing patient activation levels in pleasurable or mastery activities, and comprises the following strategies: psychoeducation, behavioural assessment, activity monitoring, activity structuring and scheduling, activation of social networks, and problem solving. HAP was delivered in an individual format and involved 6–8 sessions, each lasting 30–40 minutes, with the initial sessions being at weekly intervals. The PT consisted of three phases: a beginning phase focused on orienting to treatment, a multi-session middle phase on teaching core intervention strategies, and a late phase on reviewing gains and termination. The middle phase could be extended with up to 2 additional sessions for patients who did not show sufficient improvement, allowing a maximum of 8 sessions across all phases. Patients who did not respond by the end of treatment were referred for specialist care. Details about the intervention are reported elsewhere [13] and can be accessed online (http://hap.nextgenu.org). A description of counsellor selection, training, and supervision is published elsewhere [13,21]. Counsellors were members of the local community, were above 18 years of age, had completed at least high school education, and did not have prior professional mental health training. Counsellors underwent a 3-week participatory workshop covering both PTs (HAP and CAP), followed by an internship phase of 6 months, in which trainee counsellors delivered the treatment to eligible patients in PHCs. Eleven counsellors who met competency standards participated in the trial. They received weekly peer-led supervision in groups of 4–6 and individual supervision twice monthly.

The same counsellor delivered the HAP treatment to individuals with depression and the CAP treatment to adult males who met criteria for harmful drinking. Counsellors maintained separate clinical registers for the 2 groups of patients and reviewed individual patient records before each session. In order to ensure that their treatment-specific counselling skills were maintained throughout the trial, weekly peer-led group supervision sessions were structured in ways that involved holding separate sessions for each of the 2 treatments. This arrangement allowed the expert supervisors for each of the 2 treatments to provide more focused feedback to the counsellors.

Treatment fidelity was assessed at 2 levels: the quality with which HAP was delivered and the quantity of the dose of HAP administered. The quality of HAP was assessed based on a random selection of 10% of audio-recorded sessions, rated on a therapy quality scale [21] by peers and experts. The quantity of HAP delivered was assessed based on treatment completion records maintained by the counsellors.

### Outcomes

The 2 primary outcomes for the 12-month analyses were (1) depression severity assessed by BDI-II (dropping the item related to sex for cultural reasons) and (2) remission from depression (defined as PHQ-9 score < 10). Our PHQ-9 cutoff for remission is in alignment with the depression treatment literature, which defines remission as either the complete absence of symptoms, which is reflected by a PHQ-9 score < 5, or a partial absence of symptoms, defined as PHQ-9 score < 10 [22,23]. A range of secondary outcomes included recovery from depression (PHQ-9 score < 5 at both 3 and 12 months), relapse (partial or full), disability (WHO Disability Assessment Schedule 2.0 [WHODAS 2.0]), suicidal behaviour, and intimate partner violence.

We estimated the minimal clinically important difference (MCID) as a patient-centred metric that captures both the magnitude of improvement and the value the patient places on that improvement [24]. We used the anchor-based approach for estimating MCID that ties change in outcome on the PHQ-9 to the patient’s subjective sense of improvement [25]; patients’ rating of perceived improvement on a ‘global rating of change’ scale [26] was used to calculate the corresponding difference in score (see S1 Table for definition of all secondary outcomes). In addition, we assessed patient-reported activation levels at 3 months, using a 5-item Likert scale (0–5) based on the Behavioral Activation for Depression Scale–Short Form (BADS-SF) [27], to test for mediation. This behavioural activation variable was pre-specified as a potential mediator of HAP on depression outcomes because patient activation levels are the primary focus of treatments for depression based on the theory of behavioural activation. All measures were carefully selected based on their psychometric properties and contextual appropriateness. The BDI-II is a widely used measure for evaluating depression in trials, and has been used in surveys in India [28]. The PHQ-9 has been validated in primary care, and a Konkani (widely spoken local language in trial area) version validated in Goa [29]. WHODAS 2.0 is validated for international use and was used in previous trials in Goa [30,31]. The Client Service Receipt Inventory (CSRI), which was used to collect information on health service use for the economic evaluations, has been previously used in trials in the study setting [32,33]. The 2 items on intimate partner violence were selected based on interviews used in earlier studies in Goa [34], and the BADS-SF was translated into Konkani using standardised procedures followed by piloting [13].

### Statistical methods

Analyses were on an intention-to-treat basis using multiple imputations (20 iterations) for missing outcome data via a data augmentation algorithm in Stata 14.0. All models adjusted for baseline PHQ-9 score and for PHC as a fixed effect to allow for within-PHC clustering. For continuous outcomes, intervention effects were estimated using linear regression and are reported as AMDs or ESs with 95% CIs. For binary outcomes, intervention effects are reported as aPRs estimated from logistic regression using the marginal standardisation technique for the prevalence ratios and the delta method for the CIs [35]. Sensitivity analyses included adjustment for counsellor as a random effect and complete case analyses. Repeated measures analyses were conducted to estimate the time-by-treatment interaction effect. In addition, we examined changes in mean outcome scores over time, by treatment condition. The MCID was estimated using receiver operating characteristic (ROC) analysis in order to establish the minimum relative change in PHQ-9 score that best differentiated those individuals who felt better from those who did not. We applied the cut-point for minimum specificity of 70% suggested by Button and colleagues [25]. Following cut-point determination, a binary outcome variable was created, and intervention effects reported as aPRs estimated from logistic regression. Results are described in terms of strength of evidence rather than statistical significance; hence, we did not adjust *p*-values for multiple comparisons [36]. Our approach to the mediation analysis involved the Monte Carlo method for assessing mediation [37,38], which has been shown to be more rigorous than the Sobel test and as accurate as bootstrapping [39]. In the current study, we computed a 95% CI with 20,000 repetitions. All regression models controlled for individual patients’ baseline PHQ-9 score as well as any variables that were found to be significantly related to either the proposed mediator or 12-month BDI-II score. The variance inflation factor (VIF) was calculated for each independent variable that was entered into each regression model to assess multicollinearity between independent variables, with a conservative cutoff for defining multicollinearity (VIF ≥ 5).

Economic evaluations were conducted from both the healthcare system perspective (costs to the health system only) and the societal perspective (health system costs plus impacts on the productivity of patients and their families). Information on the use of health services, including contacts with PHCs, hospital doctor contacts and inpatient stays, medication use, and diagnostic tests, was collected from service users using a tailored version of the CSRI at 3 and 12 months. Unit costs for doctor contacts and inpatient stays were inflated to 2015 prices using unit costs that had previously been used for an economic evaluation in Goa [40]. Detailed information on medications and laboratory tests used and costs to the public purse were recorded. Mean costs were then extrapolated to cover the full 12 months. Detailed information was also recorded on the time taken to deliver each HAP session, whether delivered at a PHC, over the telephone, or at a patient’s home. Travel time and transportation costs were also recorded for home visits, including ‘no show’ home visits. Per minute unit costs for counsellors, taking account of their training, supervision, and other overheads, were then attached to time to estimate the total costs of intervention delivery.

Productivity costs consisted of patient time out of usual activities because of their health, as well as time costs for patients (and accompanying family members) related to the use of health services. The number of days completely out of normal role (i.e., days unable to work) over the previous 30 days was based on patient responses to the WHODAS 2.0 at 3 months and 12 months. WHODAS 2.0 data on days of activity cutback over this period were also included, with the assumption that each day of cutback would have half the value of a complete day out of role, an approach that has been adopted in high-income settings [41]. Patients reported how much time was spent attending health services using the CSRI; patients were also asked to report if they were accompanied by someone. If so, it was also assumed that 1 family member incurred the same level of productivity loss. We assumed that the mean of patient and family time costs at 3 months and 12 months would also apply to the rest of the year. Costs due to cutback and complete days out of role were adjusted to avoid double counting time that patients spent attending health services. All patient and family time was valued using the human capital approach making use of different daily wage rates recommended in 2015 by the Indian Office of the Labour Commissioner. The rate used was dependent on whether the patient was classified as an unskilled, skilled, or clerical/professional worker. We assumed the value of days out of role for those classified as unemployed was the same as that for unskilled workers.

QALYs were derived through transformation of WHODAS 2.0 12-item scores, as in earlier Indian trials [40]. Incremental cost-effectiveness ratios (ICERs) were bootstrapped, randomly resampling pairs of outcomes and costs for intervention and comparator groups to derive 95% CIs, with a distribution of mean incremental costs and effects shown on cost-effectiveness planes to test the robustness of cost results. Cost-effectiveness acceptability curves were also generated, showing the likelihood that HAP would be cost-effective at different levels of willingness to pay. Statistical analyses were conducted using Excel 2016 and SPSS 21 for the cost-effectiveness analyses, SAS and R-Studio for the mediation analyses, and STATA 13/14 for all other analyses. All costs are presented in 2015 international dollars (http://eppi.ioe.ac.uk/costconversion/).

## Results

### Trial conduct

**Fig 1:**
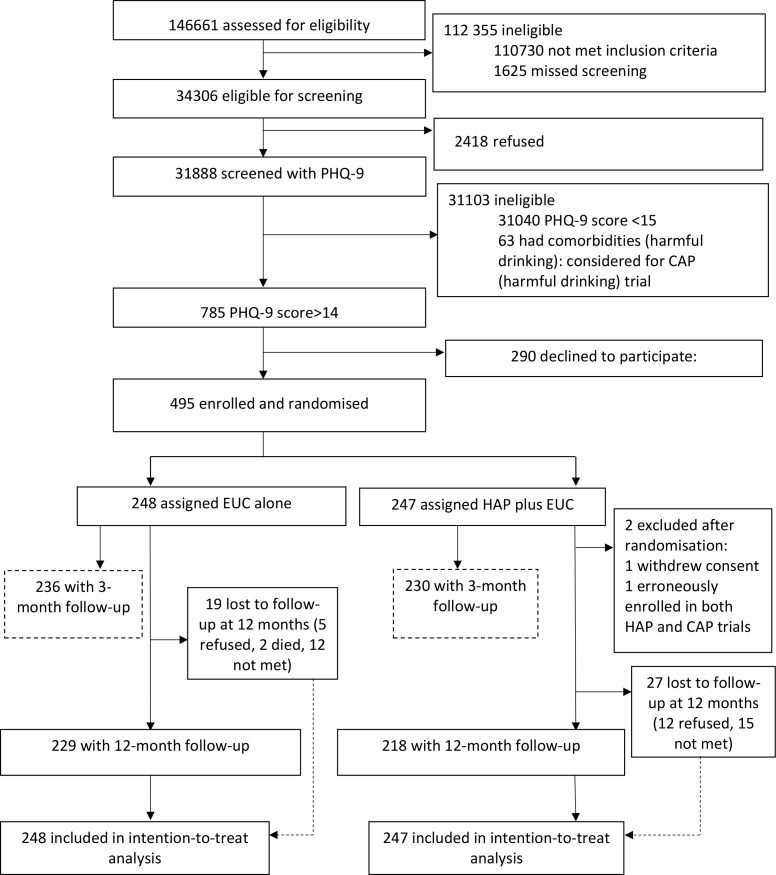
The healthy activity programme trial flow chart. CAP, Counselling for Alcohol Problems; EUC, enhanced usual care; HAP, Healthy Activity Programme; PHQ-9, Patient Health Questionnaire 9.

A detailed description of the conduct of the trial is provided in the primary trial paper [16]. Between 28 October 2013 and 29 July 2015, 34,306 (23%) of the 146,661 PHC attendees assessed met inclusion/exclusion criteria. Of these, 31,888 adult PHC attendees were screened for depression using the PHQ-9, of whom 785 (2.5%) were eligible (PHQ-9 score > 14) for inclusion in the trial, and 495 (63%) consented to participate and were enrolled. A total of 248 participants were randomised to EUC, and 247 to HAP plus EUC. Of the latter, 2 were subsequently excluded (1 withdrew consent and 1 was erroneously enrolled in both trials), leaving a total of 245 participants treated with HAP plus EUC (Fig 1). The modal reason for non-participation was lack of time, and participants had similar baseline characteristics to non-participants. Baseline characteristics were similar by arm. In all, 466 participants (95%) were assessed at the 3-month post-enrolment endpoint, and 447 participants (91%) at the 12-month follow-up; rates were similar between arms. A total of 438 (89%) participants had observations for both follow-up time points. In all, only 18 (3.6%) participants did not have any follow-up data. Those lost to follow-up at 12 months were younger (S2 Table), and this was similar at the 3-month post-enrolment endpoint. The intra-cluster correlation of BDI-II within PHCs was 0.02.

### Impact on clinical outcomes

**Fig 2:**
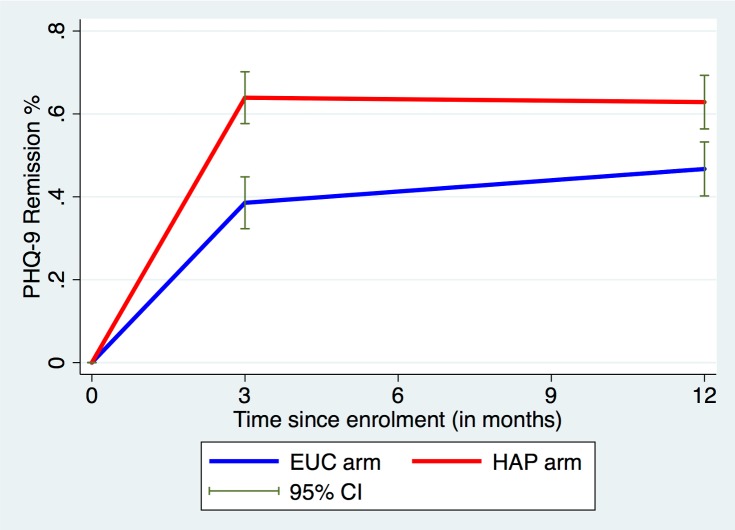
Remission rates over time in the HAP plus EUC and EUC arms. EUC, enhanced usual care; HAP, Healthy Activity Programme; PHQ-9, Patient Health Questionnaire 9.

There was an intervention effect on both primary outcomes at the 12-month follow-up. The mean endpoint BDI-II score was 19.73 (SD 15.53) among participants in the HAP plus EUC arm and 24.09 (SD 14.67) among participants in the EUC arm (AMD = −4.45; 95% CI −7.26, −1.63; ES = 0.23; 95% CI 0.18, 0.28; *p =* 0.002; Table 1). This main effect at 12 months was influenced by the passage of time (*p*-value for time-by-treatment interaction = 0.04), such that participants in the EUC arm continued to improve through the 12-month follow-up (difference in mean BDI-II score between 3 and 12 months = 3.2; 95% CI 1.34, 5.06; *p =* 0.001; S3 Table) while participants in the HAP plus EUC arm essentially retained the greater gains that they had made at the earlier assessment (difference in mean BDI-II score between 3 and 12 months = −0.34; 95% CI −2.37, 1.69; *p =* 0.74; S3 Table). Participants in the HAP plus EUC arm also had a higher probability of remission than those in the EUC arm (63% versus 48%; aPR = 1.36; 95% CI 1.15, 1.61; *p <* 0.001). As was the case for mean scores on the BDI-II, remission rates stayed relatively constant from 3 to 12 months among participants in the HAP plus EUC arm, whereas those in the EUC arm showed a slight increase by 12 months (Fig 2). Sensitivity analysis showed similar results (S4 Table). There was no evidence of moderation by sex, illness severity, duration of illness, or patient expectations (S5 Table).

**Table 1:** Effects of HAP plus EUC compared with EUC alone on primary and secondary clinical outcomes at 12 months.

Outcome	EUC arm (*n =* 248)	HAP + EUC arm (*n =* 245)	Measure: point estimate (95% CI)^1^	*p*-Value
**Primary outcomes at 12 months**				
Mean BDI-II score (SD)*	24.09 (14.67)	19.73 (15.53)	AMD: −4.45 (−7.26, −1.63); ES: 0.23 (0.18, 0.28)	0.002
Remission: PHQ-9 score < 10**	117 (46.98%)	155 (63.14%)	PR: 1.36 (1.15, 1.61); PD: 16.66% (7.85%, 25.47%)	*<*0.001; *<*0.001
**Secondary outcomes at 12 months**				
Recovery: PHQ-9 score < 5 at 3 and 12 months***	33 (13.27%)	64 (26.10%)	PR: 1.98 (1.29, 3.03); PD: 12.96% (5.31%, 20.61%)	0.002; 0.001
Full relapse: PHQ-9 score = 15–27***	12 (4.92%)	21 (8.78%)	PR: 1.79 (0.87, 3.69)	0.14
Partial relapse: PHQ-9 score = 10–14***	7 (2.70%)	21 (8.60%)	PR: 3.19 (1.27, 7.88)	0.01
Mean PHQ-9 score (SD***)	10.46 (7.54)	8.16 (6.96)	AMD: −2.36 (−3.70, −1.02); ES: 0.37 (0.32, 0.42)	*<*0.001
Any response over 12 months	266 (53.97%)	383 (77.65%)	PR: 1.45 (1.27, 1.66)	*<*0.001
Suicidal behaviour^#^	66 (26.55%)	47 (19.10%)	PR: 0.71 (0.51, 1.01)	0.06
MCID (percent reduction in baseline PHQ-9 score)^$^	102 (41.25%)	142 (58.10%)	PR: 1.42 (1.17, 1.71); PD: 17.08% (7.89%, 26.26%)	*<*0.001; *<*0.001

Data given as number (percent) unless otherwise indicated.

^1^AMD adjusted for primary health centre as a fixed effect and PHQ-9 baseline score.

*Sensitivity analysis AMD point estimate (95% CI): random effects, −4.41 (−7.21, −1.61); complete case, −4.57 (−7.34, −1.81); excluding unmasked (3.7%), −4.40 (−7.29, −1.51).

**Sensitivity analysis PR point estimate (95% CI): complete case, 1.36 (1.14, 1.61).

***Not previously specified in trials protocol but specified in published analysis plan.

^#^Suicidal thoughts over the past 2 weeks were assessed through the relevant PHQ-9 item while suicide attempts were assessed over the 3-month period leading up to the 12-month outcome follow-up assessment. Suicide attempts were not included because the numbers were very small (only 2 patients [1 in each arm] reported suicide attempt over the period).

^$^Estimated based on relative difference in baseline and outcome score, and how this compares with overall subjective global rating of ‘feeling better’ at the end of the trial. The optimal cutoff in relative change in score with maximum specificity (>70%) was 55.

AMD, adjusted mean difference; BDI-II, Beck Depression Inventory–II; ES, effect size; EUC, enhanced usual care; HAP, Healthy Activity Programme; MCID, minimal clinically important difference; PD, prevalence difference; PHQ-9, Patient Health Questionnaire 9; PR, prevalence ratio.

**Fig 3:**
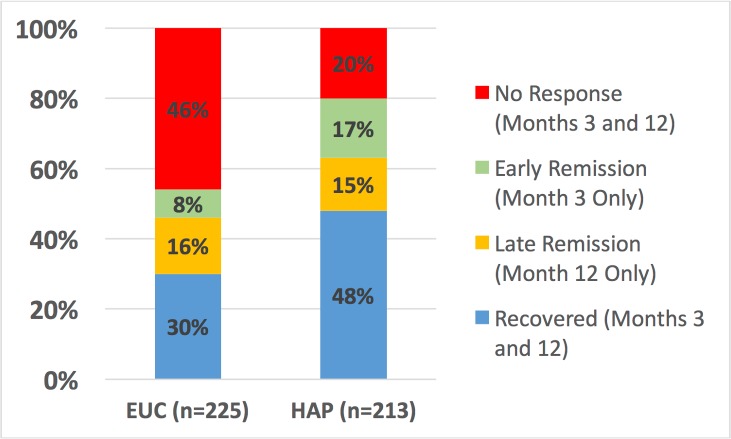
Clinical trajectories in cases with 3- and 12-month outcome data (n = 438). EUC, enhanced usual care; HAP: Healthy Activity Programme.

Participants in the HAP plus EUC arm had a higher probability of remission and recovery compared to those in the EUC arm (Table 1). While participants in the HAP plus EUC arm who had remitted at 3 months had a higher probability of partial relapse at 12 months compared to those in the EUC arm, the proportion with full relapse was similar between arms (Table 1). Participants in the HAP plus EUC arm also had a higher probability of any response over the 12 months (Table 1; Fig 3). More participants remitted in the HAP plus EUC arm in the short term compared to the EUC alone arm, but, as expected, participants who remitted in the HAP plus EUC arm were more likely to relapse following treatment termination than patients who remitted in the EUC alone arm (Fig 3). Participants in the HAP plus EUC arm had marginally lower prevalence of suicidal behaviour (mainly suicide thoughts as there were only 2 suicide attempts) at 12 months. Our analysis of what constitutes a MCID yielded a relative score change of 55% from baseline. Based on this score change, HAP plus EUC was superior to EUC at 12 months (aPR = 1.42; 95% CI 1.17, 1.71; *p <* 0.001; Table 1).

### Other outcomes and mediation analyses

**Fig 4:**
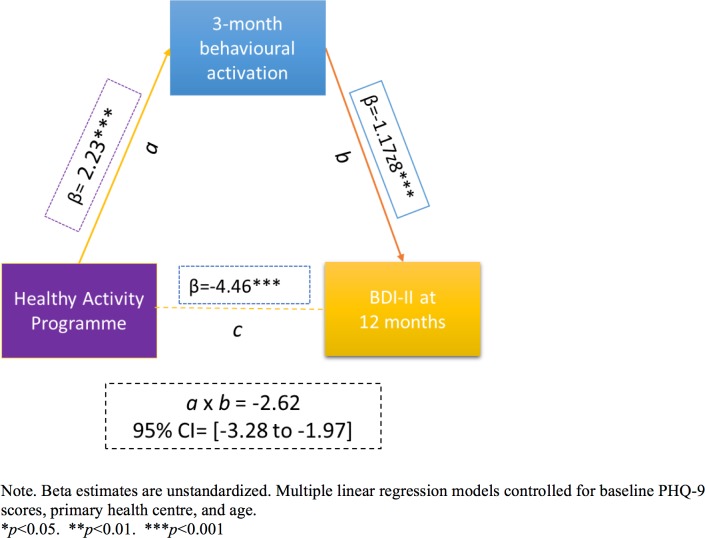
The mediating effect of behavioural activation at 3 months on the effectiveness of the HAP on depression severity at 12 months. Variables as follows: β, Beta coefficient; a, a-path (HAP–mediator); b, b-path (mediator–outcome); c, direct effect (HAP–outcome); a × b, indirect effect. BDI-II, Beck Depression Inventory–II; PHQ-9, Patient Health Questionnaire 9.

HAP plus EUC had a marginal effect on WHODAS 2.0 score at 12 months (aPR = −1.58; 95% CI −3.33, 0.17; *p =* 0.08); other outcomes (days unable to work, intimate partner violence toward females) did not statistically significantly differ between the two arms (Table 2). The prevalence of serious adverse events (HAP plus EUC arm, 23; EUC arm, 23) and proportion of participants prescribed antidepressant medications (ADMs) (HAP plus EUC arm, 7; EUC arm, 11) did not differ between the treatments (S6 Table). Our assessment of mediation demonstrated that patient-reported behavioural activation level at 3 months partially mediated the superiority of HAP plus EUC relative to EUC in terms of reduced depression severity at 12 months (β = −2.62; 95% CI −3.28, −1.97; *p <* 0.001; Fig 4; also S7 Table). Patient-reported behavioural activation could account for 58% of the total effect of HAP plus EUC. None of the models evidenced multicollinearity between the independent variables (VIF < 5).

**Table 2:** Effect of HAP plus EUC compared with EUC alone on disability and intimate partner violence at 12 months.

Outcome	EUC arm (*n =* 248)	HAP + EUC arm (*n =* 245)	Measure: point estimate (95% CI)^1^	*p-*Value
Mean disability score (SD)	10.89 (9.22)	9.38 (9.61)	AMD: −1.58 (−3.33, 0.17); ES: 0.16 (0.12, 0.19)	0.08
Mean days unable to work (SD)	6.05 (8.81)	4.81 (8.24)	AMD: −1.29 (−2.89, 0.31); ES: 0.15 (0.11, 0.19)	0.12
Intimate partner physical violence*—females	20/118 (16.57%)	11/109 (9.86%)	PR: 0.60 (0.29, 1.22)	0.16
Intimate partner psychological/emotional violence*—females	40/118 (33.86%)	28/109 (26.10%)	PR: 0.75 (0.50, 1.13)	0.17
Intimate partner psychological/emotional violence*—males	12/40 (28.75%)	7/34 (19.23%)	PR: 0.82 (0.36, 1.84)	0.62

Data given as number (percent) unless otherwise indicated.

^1^AMD adjusted for primary health centre as a fixed effect and Patient Health Questionnaire 9 baseline score.

*Among married participants.

AMD, adjusted mean difference; ES, effect size; EUC, enhanced usual care; HAP: Healthy Activity Programme; PR, prevalence ratio.

Of the 245 participants in the HAP plus EUC arm (receiving a total of 1,181 HAP sessions), 169 (69%) had a planned discharge, of whom 7 (4%) were referred for specialist care. The median number of sessions was 6 (IQR 5 to 7). Patients with an unplanned discharge were likely to stop attending early (median 1 session [IQR 0 to 2]).

### Costs and cost-effectiveness

**Fig 5:**
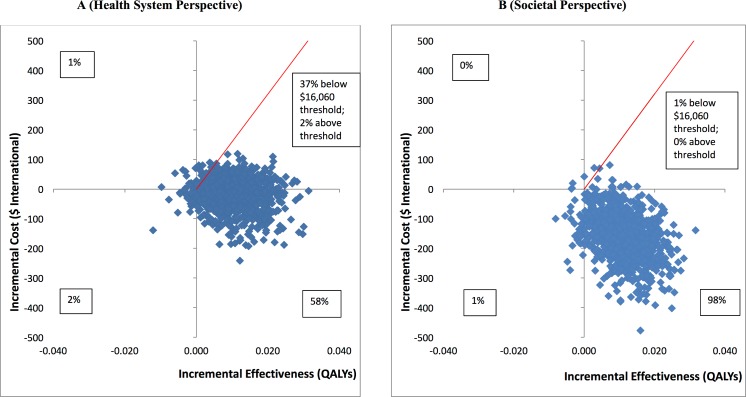
Cost-effectiveness planes: HAP plus EUC compared to EUC. (A) Health system perspective; (B) societal perspective. EUC, enhanced usual care; HAP, Healthy Activity Programme; QALY, quality-adjusted life year.

While the health system costs of HAP + EUC were significantly higher than those of EUC alone at 3-month follow-up due to the cost of providing HAP, by 12 months these costs were offset by reductions in the use of health services through month 12, and there was no statistically significant difference in health system costs between the 2 arms (S8 Table). From a wider societal perspective, which combines impacts on the health system with impacts on productivity costs, the HAP plus EUC arm had significantly lower costs at 12 months (mean difference = −$154.93; 95% CI −$305.51, −$4.35; *p =* 0.044). This was due to lower costs of days out of work and work cutback (mean difference = −$146.28; 95% CI −$218.08, −$74.47; *p* < 0.001). While there is still a gain in mean QALYs per person at 12 months compared to at 3 months, this difference was not quite statistically significant (mean difference = 0.011; 95% CI 0.006, −0.002; *p =* 0.092). Table 3 provides an assessment of cost-effectiveness showing ICERs. It indicates that the incremental cost per QALY gained is −$1,721; thus, HAP plus EUC is associated with both lower costs and better outcomes than EUC alone. To test the robustness of the ICER results, 2 cost-effectiveness analysis planes were generated using 1,000 randomly resampled pairs of costs and QALY outcomes from both the health system and societal perspectives to generate further values of incremental cost per QALY gained (Fig 5). This can help policymakers by showing the likelihood that any intervention will be cost-effective or even cost-saving. Fig 5A indicates that HAP plus EUC has a 58% chance of being cost-saving from a health system perspective, i.e., 58% of the 1,000 pairs of costs and QALYs are in the southeast quadrant, which indicates that the intervention (in this case HAP plus EUC) has both lower costs and better QALY outcomes than EUC alone, while a further 39% of the 1,000 pairs of costs and QALYs fall in the northeast quadrant, where HAP plus EUC is more effective but more expensive than EUC alone. Nearly all of the observations in this quadrant were still below the cost-effectiveness threshold used in the analysis (shown by the red line) of GDP per capita per additional QALY gained, a threshold which has been applied in economic evaluations in LMICs [42]. This threshold in the state of Goa expressed in international dollars in 2015 was $16,060 [43]. Overall, this means that the case for investment is very strong, with a 95% likelihood that investment in the intervention will be cost-effective, including a 58% chance that it will be cost-saving. Similarly, in Fig 5B when costs also include a conservative estimate of productivity losses to patients and families, 98% of the pairs of costs and QALYs fall in the southeast quadrant, where HAP plus EUC is cost-saving with lower costs and better outcomes compared to EUC alone. As Table 3 shows, if the same approach is used to look at costs per additional remission achieved compared to EUC from a health system perspective, HAP plus EUC would be considered a highly worthwhile investment (S2 Fig), with a 90% chance of being cost-effective, including a 59% chance of being cost-saving.

**Table 3:** Cost-effectiveness analyses from health system and societal perspectives (costs in 2015 international dollars).

Category	Health system perspective		Societal perspective	
	Cost (95% CI)	Likelihood ICER is CS and CE	Cost (95% CI)	Likelihood ICER is CS and CE
Per QALY gained at 12 months*	−1,721 (−23,966, 18,158)	CS: 58%; CE: 95%	−14,438 (−81,359, 13,966)	CS: 98%; CE: 99%
Per remission at 12 months**	−149 (−1,304, 988)	CS: 59%; CE: 90%	−1,250 (−3,869, −186)	CS: 99%; CE: 100%

*Assumes willingness to pay threshold equivalent to GDP per capita in Goa ($16,060).

**Assumes willingness to pay threshold equivalent to 1 month’s wages for unskilled manual worker in Goa ($415).

CE, cost-effective; CS, cost-saving; ICER, incremental cost-effectiveness ratio; QALY, quality-adjusted life year.

## Discussion

We report on the sustained effects, the cost-effectiveness, and the role of behavioural activation in mediating the effectiveness of HAP, a brief PT delivered by lay counsellors to primary care attendees with moderately severe to severe depression in a randomised controlled trial in India. We have 2 main findings.

Our first main finding was that the effects of HAP on acute depression observed shortly after the end of treatment (3 months post-enrolment) were largely sustained through the 12-month follow-up. This is striking because depression tends to return after treatment termination among recently remitted patients, which is one of the reasons physicians are encouraged to keep patients on active medications for at least 4 months following initial remission [23]. What makes this finding less surprising is that HAP is adapted from behavioural activation, and this approach was found to reduce the risk for subsequent relapse by more than half relative to prior medications in the one study in which they have been compared [44]. Patients who remitted on HAP in the short term were more likely to relapse following treatment termination than patients who remitted in EUC, but that is to be expected since more patients remitted on HAP than in EUC, and it is plausible that those additional remitters were patients at higher risk (Fig 3). That being said, HAP’s effects were relatively stable over time (i.e., depression severity scores did not change), and the absolute relapse rate was lower than that observed for behavioural activation in the largest comparable trials [44]. In a disorder that is prone to relapse, this finding augers well for the possibility that HAP might have an enduring effect.

Our second major finding was that HAP essentially pays for itself and more. It cost $65.66 per patient to provide HAP, but that extra treatment cost was completely offset by reductions in other healthcare expenses over the course of a year, so that healthcare costs between the 2 trial arms were no longer significantly different at 12 months (they had been significantly higher for HAP plus EUC in the 3-month analysis [16]). Moreover, there was a very high probability (95%) of HAP plus EUC being cost-effective from a health system perspective, including a 58% probability that it would be costsaving. What our data suggest therefore is that the initial additional costs of providing HAP will be at least budget neutral from a health system perspective, while improving clinical outcomes.

When we factor in societal costs in terms of productivity, the economic benefits of HAP become even more evident. Poor mental health has been associated with significantly lower rates of participation in employment in low-, middle-, and high-income countries, including in India, where severe mental illness has been associated with a 40% reduction in individual earnings [45]. Poor mental health also reduces the opportunity to contribute in other ways to the economy, such as household activities; it also increases the use of informal care and support from families. Our analysis also indicates that major gains are made in terms of productivity that have real implications for the individuals involved and for the larger society in which they are embedded. The United Kingdom has committed over £700 million to train therapists to deliver empirically supported treatments like behavioural activation on the premise that doing so would be good for the economy [46]. Our data suggest that this assumption might well hold for this Indian setting, despite the substantial structural differences that mean that the interventions and their contexts are not directly comparable.

Additionally, we observed that patients who received HAP reported feeling better subjectively at 12 months post-enrolment than patients who received EUC alone. Not only were HAP patients better in terms of reported symptoms, but they had the subjective sense that they were better in ways that actually mattered to them. This adds a patient-centred outcome to our main effectiveness results. At the same time, our mediation analysis suggested that patient-reported levels of behavioural activation at 3 months mediated the effects of HAP in reducing depression severity at 12 months. This suggests that behavioural activation may underlie HAP’s sustained effects and, thus, adds to existing evidence suggesting that patient-reported activation levels mediate response to behavioural activation therapy as specified by theory [47,48].

Our effects were modest and about a third of patients treated with HAP remained at least moderately symptomatic. That being said, HAP was a very brief treatment by western standards (only 6–8 sessions) and was delivered by lay counsellors; most efficacy trials provide 2–3 times that many sessions delivered by highly trained professionals [49,50]. Treatment differences did narrow over time from the 3-month post-enrolment assessment to the 12-month follow-up, but that was largely a function of continued improvement in the EUC arm (likely due to spontaneous remission) and not any loss of efficacy for HAP over time (within-condition changes were not significant). Even the elevated relapse rate for HAP plus EUC relative to EUC alone was limited to partial relapse (requiring a change of as little as a point to rise to 10 or above on the PHQ-9); there were no differences with respect to full relapse (scores of 15 or above). Notwithstanding these notable benefits, it is clear that HAP is not sufficient as a stand-alone treatment for depression for a sizeable minority of patients in primary care. Whether its dosage or duration needs to be extended or non-responders switched to or augmented with another treatment (like medications) remains to be determined.

We acknowledge limitations of this study design. First, from a methodological perspective, we had only 2 assessment time points, at 3 months and 12 months, thus precluding detection of possible episodes of remission and relapse between these 2 time points [51]. Second, we continue to observe a pattern of discordance between our 2 primary outcome measures at 12 months similar to what we found in our 3-month outcome assessments: patients at 12 months were at the low end of the moderate range of severity on the BDI-II, but the same patients were indicated as having mild residual symptoms on the PHQ-9. This suggests potential cross-cultural challenges with the use of the BDI-II, which we are currently investigating in a separate report. Third, and according to the sequential ignorability assumption [52], there is a chance that there may be other confounders that we did not assess that may explain the relation between the proposed mediator (in this case, patient activation) and depression outcomes. While our proposed mediator was selected a priori and was based on the conceptual theory of behavioural activation, future studies considering additional mediators through, for example, comprehensive structural equation models are required to verify our findings and address the sequential ignorability assumption [53]. Lastly, we did not apply diagnostic criteria in recruiting patients at baseline or in our definition of outcome, but we note that the PHQ-9 is widely used to define case-level morbidity in trials and, importantly, we used locally validated cutoffs in this study [29].

### Clinical implications and conclusions

In conclusion, our findings are consistent with the small but growing body of evidence suggesting an enduring effect for behavioural activation or more cognitive behavioural approaches [44,50,54]. HAP is unique in that, despite its brevity and delivery by a lay counsellor, it is able to sustain short-term gains in a primary care setting in a lower-middle-income country. In addition, HAP is only 1 of 2 [55] brief PTs based on behavioural activation theory delivered by lay counsellors in primary care settings yet evaluated. The low levels of ADM use noted in our study, even after the diagnosis was conveyed to the primary care physician, confirms that the effect of HAP could not have been confounded by ADM use, and further supports the applicability of the HAP treatment in this treatment-naïve population. The ecological validity of the trial was enhanced by the fact that the lay counsellors had no prior professional mental health training (as would be the case in most real-world settings) and that they were concurrently delivering a completely different PT for harmful drinking (as would be the case in actual practice) [56]. The importance of establishing sustained effects of treatments cannot be overemphasised given that depression tends to relapse or recur. We have demonstrated that brief PTs like HAP and CAP delivered by non-specialist mental health workers in routine primary care can have sustained clinical effects and are good value for the money. Such treatments are ideal for scaling up, and future research should focus on (1) employing Sequential Multiple Assignment Randomized Trial (SMART) designs to assess how different interventions can be applied in sequence to achieve higher rates of remission and recovery [57] and (2) examining the potential roles of multiple mediators within randomised trial designs so that the effectiveness of treatments can be enhanced through a focus on these mediators.

## Supporting information

S1 CONSORT Checklist (DOC)

S1 Fig Cost-effectiveness acceptability curve: Willingness to pay per QALY gained from HAP from a health system perspective. (TIF)

S2 Fig Cost-effectiveness planes: HAP plus EUC compared to EUC per remission achieved. (A) Health system perspective; (B) societal perspective.
(TIF)

S1 Protocol Study protocol. (PDF)

S1 Table Secondary outcomes at 12 months. (DOCX)

S2 Table Comparison of participants who were followed up and those lost to follow-up at 3 and 12 months. (DOCX)

S3 Table Results of *t* test and descriptive statistics for change in mean primary outcome score between the 3- and 12-month endpoints by trial arm (complete case *n =* 438). (DOCX)

S4 Table Effect of HAP plus EUC on scores for depression symptoms, disability, suicide behaviour, and intimate partner violence over 9 months, based on complete case and random effects. ^1^Adjusted for PHC as a fixed effect and PHQ-9 baseline score. ***Not previously specified in trials protocol but specified in published analysis plan. ^#^Suicidal thoughts over the past 2 weeks were assessed through the relevant PHQ-9 item while suicide attempts were assessed over the 3-month period leading up to the 12-month outcome follow-up assessment. Suicide attempts were not included because the numbers were very small (only 2 patients [1 in each arm] reported suicide attempt over the period). ^##^Among married participants. ^$^Minimal clinically important difference: estimated based on the relative difference in baseline and outcome score, and how this compares with overall subjective global rating of ‘feeling better’ at the end of the trial. The optimal cutoff in relative change in score with maximum specificity (>70%) was 55%.
(DOCX)

S5 Table Interaction effect of baseline depression severity, sex, length of depression, and expectations of treatment on the effect of HAP plus EUC on scores for depression symptoms (BDI-II outcome). ^1^Adjusted for PHC as a fixed effect and PHQ-9 baseline score.
(DOCX)

S6 Table Serious adverse events and medication use by arm in the last 3 months. (DOCX)

S7 Table Mediation results examining the effect of patient-reported activation levels at 3 months on 12-month depression outcomes. *Beta estimates are unstandardised. Multiple linear regression models controlled for baseline PHQ-9 score, participant age, and PHC. *p < 0.05. **p < 0.01. ***p < 0.001. *c*′, total effect; *a* × *b*, indirect effect.
(DOCX)

S8 Table Mean costs (2015 international dollars) and QALYs gained per person over 12 months. (DOCX)

S1 Text Statistical analysis plan. (DOC)

S2 Text HAP manual. (PDF)

## Abbreviations

ADM: antidepressant medication
AMD: adjusted mean difference
aPR: adjusted prevalence ratio
BADS-SF: Behavioral Activation for Depression Scale–Short Form
BDI-II: Beck Depression Inventory–II
CAP: Counselling for Alcohol Problems
CE: cost-effective
CS: cost-saving
CSRI: Client Service Receipt Inventory
ES: effect size
EUC: enhanced usual care
HAP: Healthy Activity Programme
ICER: incremental cost-effectiveness ratio
LMICs: low- and middle-income countries
MCID: minimal clinically important difference
PD: prevalence difference
PHC: primary health centre
PHQ-9: Patient Health Questionnaire 9
PR: prevalence ratio
PREMIUM: PRogram for Effective Mental health Interventions in Under-resourced health systeMs
PT: psychological treatment
QALY: quality-adjusted life year
VIF: variance inflation factor
WHODAS 2.0: WHO Disability Assessment Schedule 2.0
