# Supplementary figures and images for: Sustained effectiveness and cost-effectiveness of the Healthy Activity Programme, a brief psychological treatment for depression delivered by lay counsellors in primary care: 12-month follow-up of a randomised controlled trial
Source: PLoS Med. 2017 Sep 12;14(9):e1002385. doi: 10.1371/journal.pmed.1002385 (PMC5595303; doi:10.1371/journal.pmed.1002385)

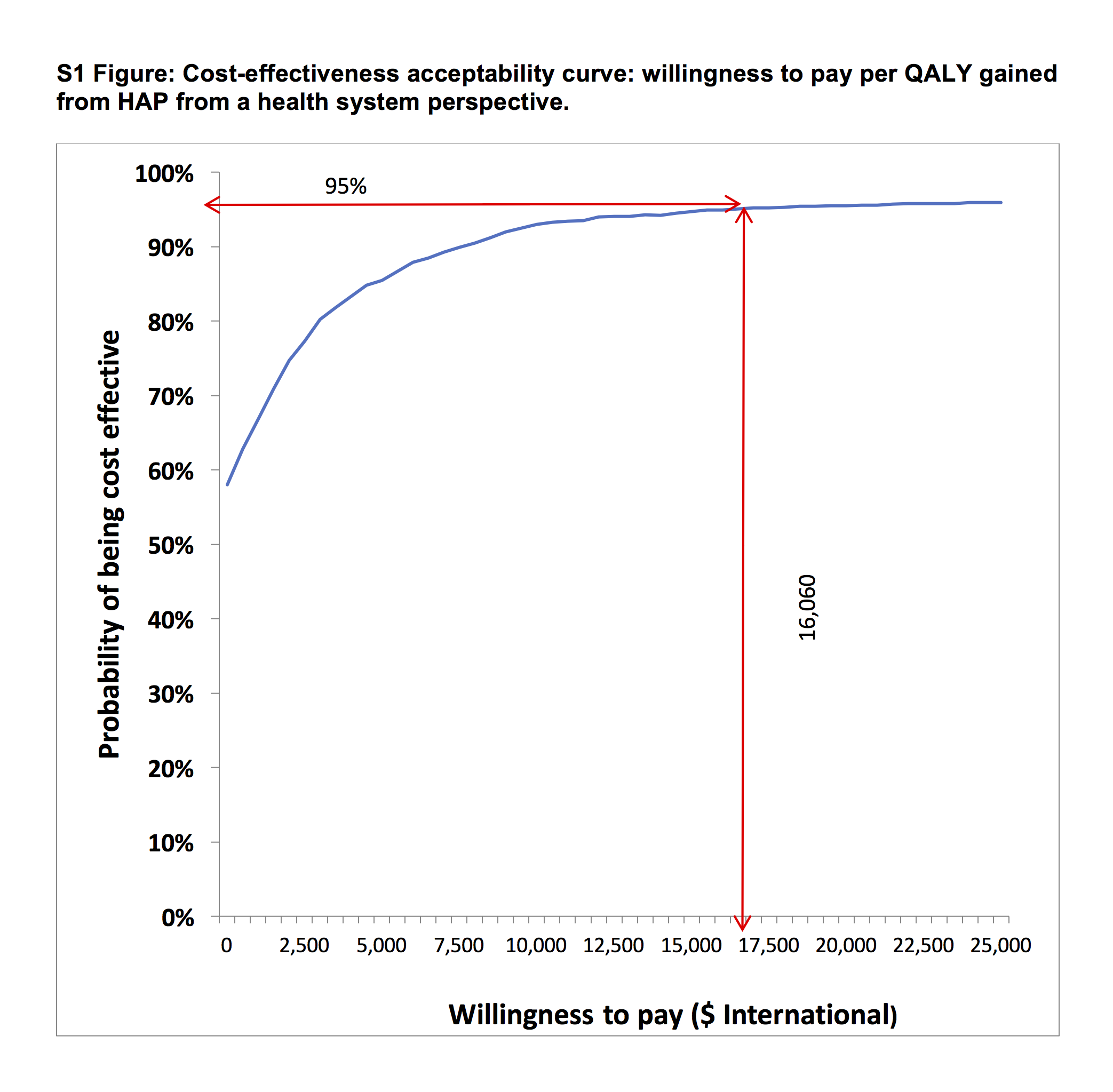

Supplement: S1 Fig — (TIF) [file pmed.1002385.s002.tif]

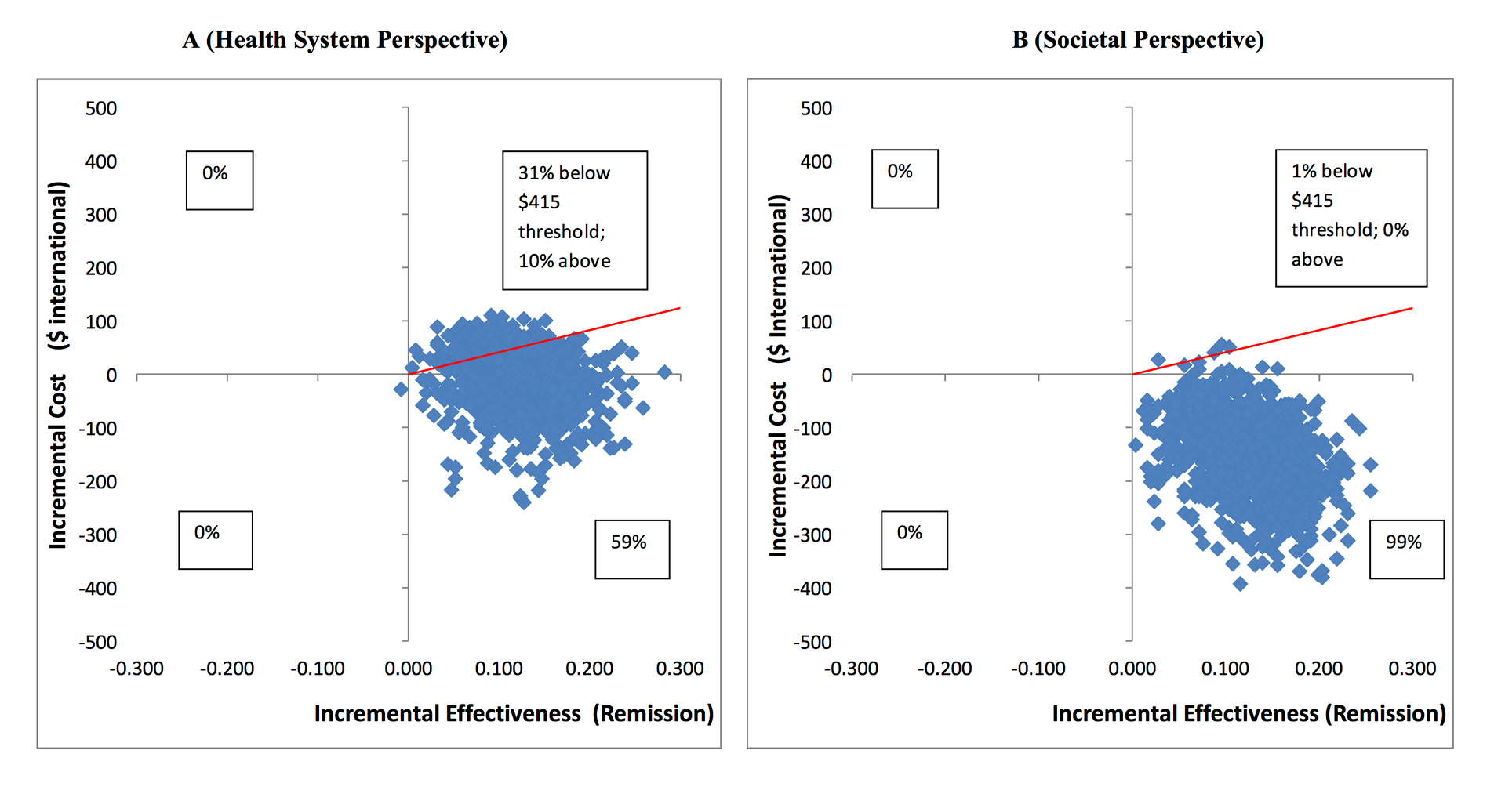

Supplement: S2 Fig — (A) Health system perspective; (B) societal perspective. (TIF) [file pmed.1002385.s003.tif]
