# Supplementary material for: Sustained effectiveness and cost-effectiveness of the Healthy Activity Programme, a brief psychological treatment for depression delivered by lay counsellors in primary care: 12-month follow-up of a randomised controlled trial
Source: PLoS Med. 2017 Sep 12;14(9):e1002385. doi: 10.1371/journal.pmed.1002385 (PMC5595303; doi:10.1371/journal.pmed.1002385)
Supplement: S1 Table — (DOCX) [file pmed.1002385.s005.docx]

| **Secondary outcome** | **Measure of outcome** |
| --- | --- |
| Recovery from depression | PHQ-9 score <5 at both 3 and 12 months |
| Full relapse | PHQ-9 score >14 at 12 months amongst patients with at least partial remission at 3 months. |
| Partial relapse | PHQ-9 score 10 to 14 at 12 months amongst patients with at least partial remission at 3 months. |
| Disability | Mean disability score on the WHO disability assessment schedule version 2 (WHO-DAS II[59]) |
| Total days unable to work | Mean total days unable to work in the previous month on the WHO-DAS II. |
| Suicidal behaviour | Proportion reporting suicide thoughts in the last two weeks on the PHQ-9; proportion reporting any suicide attempts in the last 3 months |
| Intimate partner violence | Proportion reporting experience of intimate partner violence (physical/psychological/emotional) over the past 3 months. |
| Minimal Clinically Important Difference (MCID) | Change in PHQ-9 outcome score from baseline compared with the corresponding score on patient’s subjective sense of improvement. |
| Any response over 12 months | PHQ-9 score <10 at either of the 3- and 12-month outcome assessment points (added post-hoc but before analysing the data). |
| Resource impacts for the health system | Estimates of cost-effectiveness/cost-saving using detailed electronic records on HAP delivery, as well as other use of primary and secondary care services collected from patients using the Client Service Receipt Inventory[60]. |
