# Supplementary material for: Sustained effectiveness and cost-effectiveness of the Healthy Activity Programme, a brief psychological treatment for depression delivered by lay counsellors in primary care: 12-month follow-up of a randomised controlled trial
Source: PLoS Med. 2017 Sep 12;14(9):e1002385. doi: 10.1371/journal.pmed.1002385 (PMC5595303; doi:10.1371/journal.pmed.1002385)
Supplement: S2 Table — (DOCX) [file pmed.1002385.s006.docx]

|  | **Lost before 3-month evaluation N=27**  **(5.5%)** | **Completed 3-month outcome evaluation N=466 (94.5%)** | **Lost before 12-month evaluation**  **N=46 (9.3%)** | **Completed 12-month outcome evaluation N=447 (90.7%)** | **p-value (12-month follow up)** |
| --- | --- | --- | --- | --- | --- |
| *Age (years) (mean [SD]) | 36.2 (11.6) | 42.9 (12.0) | 35 (11.8) | 43 (11.8) | p<0.001 |
| **Gender** (Female) (n [%]) | 23 (85%) | 356 (76%) | 35 (76%) | 344 (77%) | p=0.86 |
| **Marital status** (n [%])  Married  Single  Separated/Divorced  Widowed | 16 (59.3%)  8 (29.6%)  1 (3.7%)  2 (7.4%) | 321 (68.9%)  49 (10.5%)  3 (0.6%)  93 (20.0%) | 33 (71.7%)  11 (23.9%)  0 (0.0%)  2 (4.4%) | 304 (68.0%)  46 (10.3%)  4 (0.9%)  93 (20.8%) | p=0.004 |
| **Education status** (n [%])  None  Primary  Secondary  Higher Secondary  Graduate/above | 6 (22%)  13 (48%)  5 (19%)  2 (7%)  1 (4%) | 124 (27%)  236 (50%)  73 (16%)  22 (5%)  11 (2%) | 7 (15%)  23 (50%)  6 (13%)  6 (13%)  4 (9%) | 123 (28%)  226 (51%)  72 (16%)  18 (4%)  8 (2%) | p=0.004 |
| **Occupation** (n [%])  Unemployed  Unskilled manual labour  Skilled manual labour  Clerical & professional | 14 (52%)  12 (41%)  0 (0%)  1 (3%) | 278 (60%)  162 (35%)  7 (2%)  19 (4%) | 22 (48%)  18 (39%)  0 (0%)  6 (13%) | 270 (60%)  156 (35%)  7 (2%)  14 (3%) | p=0.02 |
| **Patient’s expectation of counselling** (n [%])  Not useful  A little/somewhat useful  Moderately useful  Very useful | 0 (0%)  8 (31%)  8 (28%)  11 (38%) | 1 (0.2%)  218 (47%)  108 (23%)  139 (30%) | 0 (0%)  14 (30%)  13 (28%)  19 (41%) | 1 (0.2%)  212 (47%)  103 (23%)  131 (29%) | p=0.13 |
| **Chronicity of symptoms-wks** (median [IQR]) | 4 (4-15) | 12 (4-48) | 9 (4-24) | 12 (4-48) | p=0.22 |
| **Median PHQ score** (median [IQR]) | 17 (15-18) | 17 (16-20) | 18 (15-19) | 17 (16-20) | p=0.55 |
| **Mean PHQ-score (SD)** | 17.3 (1.9) | 18.0 (2.8) | 17.6 (2.4) | 17.9 (2.7) | p=0.43 |
| **PHQ category** (n [%])  Score 15-19 (Mod. severe)  Score 20-27 (severe) | 24 (90%)  3 (10%) | 348 (75%)  118 (25%) | 37 (80%)  9 (20%) | 335 (75%)  112 (25%) | p=0.48 |
| **Trial Arm**  EUC  HAP+EUC | 12 (44%)  15 (56%) | 236(49%)  230 (51%) | 19 (41%)  27 (59%) | 229 (51%)  218 (49%) | p=0.22 |

* Single independent predictor of lost to follow up in multivariate analysis
