# Supplementary material for: Sustained effectiveness and cost-effectiveness of the Healthy Activity Programme, a brief psychological treatment for depression delivered by lay counsellors in primary care: 12-month follow-up of a randomised controlled trial
Source: PLoS Med. 2017 Sep 12;14(9):e1002385. doi: 10.1371/journal.pmed.1002385 (PMC5595303; doi:10.1371/journal.pmed.1002385)
Supplement: S3 Table — (DOCX) [file pmed.1002385.s007.docx]

| **Trial arm** | **Endpoint** | | **95% CI for Mean Difference** | **t** | **df** | **p-value** |
| --- | --- | --- | --- | --- | --- | --- |
|  | 3 months Mean (SD) | 12 months Mean (SD) |  |  |  |  |
| **BDI-II** |  | | | | | |
| EUC | 27.66 (13.27) | 24.46 (14.66) | 3.2 (1.34, 5.06) | 3.39 | 224 | p=0.001 |
| HAP+EUC | 19.64 (15.45) | 19.97 (15.59) | -0.34 (-.2.37, 1.69) | -0.33 | 212 | p=0.74 |
