# Supplementary material for: Sustained effectiveness and cost-effectiveness of the Healthy Activity Programme, a brief psychological treatment for depression delivered by lay counsellors in primary care: 12-month follow-up of a randomised controlled trial
Source: PLoS Med. 2017 Sep 12;14(9):e1002385. doi: 10.1371/journal.pmed.1002385 (PMC5595303; doi:10.1371/journal.pmed.1002385)
Supplement: S4 Table — 1Adjusted for PHC as a fixed effect and PHQ-9 baseline score. ***Not previously specified in trials protocol but specified in published analysis plan. #Suicidal thoughts over the past 2 weeks were assessed through the relevant PHQ-9 item while suicide attempts were assessed over the 3-month period leading up to the 12-month outcome follow-up assessment. Suicide attempts were not included because the numbers were very small (only 2 patients [1 in each arm] reported suicide attempt over the period). ##Among married participants. $Minimal clinically important difference: estimated based on the relative difference in baseline and outcome score, and how this compares with overall subjective global rating of ‘feeling better’ at the end of the trial. The optimal cutoff in relative change in score with maximum specificity (>70%) was 55%. (DOCX) [file pmed.1002385.s008.docx]

| **SENSITIVITY ANALYSIS** | **EUC arm**  **(n=229)** | **HAP+EUC arm**  **(n=218)** | **^1^Adjusted mean difference (AMD), effect size (ER), prevalence ratio (PR), prevalence difference (PD) (95% CI)** | **p-value** |
| --- | --- | --- | --- | --- |
| **PRIMARY OUTCOMES** |  | | | |
| **Complete case** |  | | | |
| Mean BDI-II score (SD) | 24.37 (14.65) | 19.83 (15.56) | AMD: -4.57 (-7.34, -1.81)  ES: 0.30 (0.11-0.48) | p=0.001 |
| Remission: PHQ-9<10- no. (%) | 107 (46.72%) | 137 (62.84%) | PR: 1.36 (1.14, 1.61) | p=0.0004 |
| **Random effects** |  | | | |
| Complete case adjusting for counsellor/PHC as random effect (BDI-II score) | 24.37  (14.65) | 19.83 (15.56) | AMD: -4.57(-7.34, -1.81)  ES: 0.30 (0.11, 0.48) | p=0.001 |
| Multiple imputation adjusting for counsellor/PHC as random effect (BDI-II score) | 24.09  (14.67) | 19.73 (15.53) | AMD: -4.41(-7.21, -1.61)  ES: 0.23 (0.17, 0.28) | p=0.002 |
| **SECONDARY OUTCOMES** |  | | | |
| **Complete case** |  | | | |
| Recovery: PHQ-9<5 at 3 and 12 months- no. (%) | 48 (20.96%) | 66 (30.28%) | PR: 1.44 (1.05, 1.97)  PD: 9.27% (1.43%, 17.11%) | p=0.022  p=0.021 |
| ***Full relapse: PHQ-9 score>14-  no. (%) | 11 (4.80%) | 19 (8.72%) | PR: 1.81 (0.88, 3.69) | p=0.11 |
| ***Partial relapse: PHQ-9 score>9<15-  no. (%) | 2 (2.62%) | 18 (8.26) | PR: 3.15 (1.27, 7.79) | p=0.013 |
| ***Mean PHQ-9 score (SD) | 10.57 (7.58) | 8.19  (6.91) | AMD: -2.41 (-3.72, -1.09)  ES: 0.33 (0.14, 0.51) | p<0.0001 |
| Any response over 12 months no. (%) | 129/240 (53.75) | 182/235  (77.45) | PR: 1.44 (1.26, 1.26) | p<0.0001 |
| Mean disability score (SD) | 10.89 (9.22) | 9.38  (9.61) | AMD: -1.58 (-3.33, 0.17)  ES: 0.03 (-0.03, 0.8) | p=0.08 |
| Mean days unable to work (SD) | 6.05  (8.81) | 4.81  (8.24) | AMD: -1.29 (-2.89, 0.31)  ES: 0.09 (0.04, 0.15) | p=0.12 |
| Suicidal behaviour (Suicide thoughts) – no. (%)# | 61/229 (26.64) | 41/218  (18.81) | PR: 0.70 (0.49, 0.99) | p=0.046 |
| Intimate partner physical violence##– females no. (%) | 19/116 (16.38) | 10/103  (9.71) | PR: 0.59 (0.29, 1.21) | p=0.149 |
| Intimate partner psychological/emotional violence## – females no. (%) | 39/116 (33.62) | 27/103  (26.21) | PR: 0.75 (0.49, 1.13) | p=0.173 |
| Intimate partner psychological/emotional violence## – males no. (%) | 11/39 (28.21) | 6/32  (18.75) | PR: 0.36 (0.37, 1.94) | p=0.711 |
| $MCID (% reduction in baseline PHQ-9 score) | 93 (40.61) | 125 (57.60) | PR: 1.21 (1.05, 1.39) | p=0.009 |
| **Random effects** |  | | | |
| Complete case adjusting for counsellor as random effect (PHQ-9 score) | 10.57  (7.58) | 8.19  (6.91) | AMD: -2.41 (-3.72, -1.09)  ES: 0.33 (0.14, 0.51) | p<0.0001 |
| Multiple imputation adjusting for counsellor as random effect (PHQ-9 score) | 10.46  (7.54) | 8.16  (6.96) | AMD: -2.34 (-3.67, -1.00)  ES: 0.37 (0.31, 0.42) | p<0.0001 |
| Complete case adjusting for counsellor as random effect (Mean disability score) | 11.05 (9.22) | 9.43  (9.62) | AMD: -1.64 (-3.34, 0.05)  ES: 0.17 (-0.01, 0.36) | p=0.057 |
| Multiple imputation adjusting for counsellor as random effect (Mean disability score) | 10.89 (9.22) | 9.38  (9.61) | AMD: -1.55 (-3.29, 0.19)  ES: 0.03 (-0.03, 0.08) | p=0.082 |
| Complete case adjusting for counsellor as random effect (Mean days unable to work) | 6.14  (8.83) | 4.81  (8.21) | AMD: -1.31 (-2.86, 0.23)  ES: 0.16 (-0.03, 0.34) | p=0.096 |
| Multiple imputation adjusting for counsellor as random effect (Mean days unable to work) | 6.05 (8.81) | 4.81 (8.24) | AMD: -1.26 (-2.86, 0.33)  ES: 0.09 (0.04, 0.15) | p=0.121 |
