# Supplementary material for: Sustained effectiveness and cost-effectiveness of the Healthy Activity Programme, a brief psychological treatment for depression delivered by lay counsellors in primary care: 12-month follow-up of a randomised controlled trial
Source: PLoS Med. 2017 Sep 12;14(9):e1002385. doi: 10.1371/journal.pmed.1002385 (PMC5595303; doi:10.1371/journal.pmed.1002385)
Supplement: S5 Table — 1Adjusted for PHC as a fixed effect and PHQ-9 baseline score. (DOCX) [file pmed.1002385.s009.docx]

| **Analysis** | **EUC arm**  **(n=248)** | **HAP+EUC arm**  **(n=245)** | **^1^Adjusted mean difference (95% CI)** | **p-value** |
| --- | --- | --- | --- | --- |
| **Baseline severity** |  | | p-effect modification = 0.227 | |
| Moderate | 22.77 [14.28] | 19.69 [15.72] | -3.54 (-6.85, -0.22) | p=0.037 |
| Severe | 28.59 [15.17] | 19.42 [15.04] | -8.41 (-14.29, -2.53) | p=0.006 |
|  |  | | | |
| **Gender** |  | | p-effect modification= 0.857 | |
| Males (mean score[SD]) | 24.28 [14.84] | 20.07 [13.60] | -3.64 (-9.44, 2.17) | p=0.216 |
| Females (mean score[SD]) | 24.24 [14.69] | 19.62 [16.15] | -4.90 (-8.26, -1.55) | p=0.004 |
|  |  | | | |
| **Chronicity** |  | | p-effect modification=0.181 | |
| <12 weeks | 21.76 [14.78] | 18.68 [15.28] | -2.89 (-6.87, 1.10) | p=0.155 |
| >=12 weeks | 27.59 [14.04] | 20.59 [15.74] | -6.86 (-10.86, -2.86) | p=0.001 |
|  |  | | | |
| **Expectation** |  | | p-effect modification=0.629 | |
| Not or somewhat useful | 23.70 [13.96] | 19.69 [15.28] | -4.15 (-8.11, -0.19); | p=0.040 |
| Moderate or very useful | 24.38 [15.58] | 20.00 [16.04] | -5.32 (-9.36, -1.29); | p=0.010 |

^1^ Adjusted for PHC as a fixed effect and PHQ-9 baseline score
