# Supplementary material for: Sustained effectiveness and cost-effectiveness of the Healthy Activity Programme, a brief psychological treatment for depression delivered by lay counsellors in primary care: 12-month follow-up of a randomised controlled trial
Source: PLoS Med. 2017 Sep 12;14(9):e1002385. doi: 10.1371/journal.pmed.1002385 (PMC5595303; doi:10.1371/journal.pmed.1002385)
Supplement: S6 Table — (DOCX) [file pmed.1002385.s010.docx]

| **SAE/psychotropic medication** | **EUC**  **number of SAEs (No. of participants)** | **HAP+EUC**  **Number of SAEs (No. of participants)** | **p-value** |
| --- | --- | --- | --- |
| **SAEs** |  | | |
| Total SAEs | 29 (34) | 17 (18) | p=0.12 |
| Death | 2 (2) | 0 (0) | p=0.49 |
| Suicide attempt | 1 (1) | 1 (1) | p=1.00 |
| Unplanned hospitalisation | 26 (31) | 18 (17) | p=0.26 |
| **Psychotropic medication** | **No. of participants** | **No. of participants** |  |
| Total psychotropic medication use | 11 | 7 | p=0.47 |
