# Supplementary material for: Sustained effectiveness and cost-effectiveness of the Healthy Activity Programme, a brief psychological treatment for depression delivered by lay counsellors in primary care: 12-month follow-up of a randomised controlled trial
Source: PLoS Med. 2017 Sep 12;14(9):e1002385. doi: 10.1371/journal.pmed.1002385 (PMC5595303; doi:10.1371/journal.pmed.1002385)
Supplement: S7 Table — *Beta estimates are unstandardised. Multiple linear regression models controlled for baseline PHQ-9 score, participant age, and PHC. *p < 0.05. **p < 0.01. ***p < 0.001. c′, total effect; a × b, indirect effect. (DOCX) [file pmed.1002385.s011.docx]

| **Assessment point** | **BA score (imputed data)** | | **Model** | **Regression Result** | | **Bootstrap 95% CI** |
| --- | --- | --- | --- | --- | --- | --- |
|  | EUC  (n=248) | HAP+EUC  (n=245) |  | Β* | SE |  |
| 3 months  mean (SD) | 9.81 (4.31) | 12.01 (4.71) | *c*’ (HAP+EUC 🡪 BDI-II at 12-months) | -4.46*** | 0.79 | (-6.01, -2.91) |
| 12 months | 10.02 (4.64) | 11.00 (4.49) | *a*’ (HAP+EUC 🡪 activation at 3-months) | 2.23*** | 0.23 | (1.77, 2.68) |
|  | | | *b*’ (activation at 3-months 🡪 BDI-II at 12 months) | -1.17*** | 0.09 | -1.35, -1.00) |
|  |  |  | a x b | -2.62*** | 0.33 | (-3.28, -1.97) |

Note: *Beta estimates are unstandardized. Multiple linear regression models controlled for baseline PHQ-9 scores, participant age, and PHC). **p*<0.05. ***p*<0.01. ****p*<0.001

c^’^ total effect; a x b: indirect effect
