# Supplementary material for: Sustained effectiveness and cost-effectiveness of the Healthy Activity Programme, a brief psychological treatment for depression delivered by lay counsellors in primary care: 12-month follow-up of a randomised controlled trial
Source: PLoS Med. 2017 Sep 12;14(9):e1002385. doi: 10.1371/journal.pmed.1002385 (PMC5595303; doi:10.1371/journal.pmed.1002385)
Supplement: S8 Table — (DOCX) [file pmed.1002385.s012.docx]

| **Type of Cost** | **HAP+EUC arm**  **(n=245)** | **EUC arm (n=248)** | **Mean Difference**  **(95% CI)** | **p-value** |
| --- | --- | --- | --- | --- |
| **HAP intervention costs** | | | | |
| HAP Intervention (SE) | 65.66 (3.48) | 0 (0) | 65.66 (58.80, 72.52) | 0.000 |
| **Health Service Utilisation** | | | | |
| PHC Doctor Consultations (SE) | 51.64 (3.86) | 58.77 (6.23) | -7.13 (-21.54, 7.28) | 0.331 |
| Hospital Doctor Consultations (SE) | 84.06 (14.51) | 116.96 (47.55) | -32.90 (-130.75, 64.95) | 0.509 |
| Hospital Admissions (SE) | 19.92 (5.32) | 39.08 (9.12) | -19.16 (-39.92, 1.60) | 0.070 |
| Laboratory Tests (SE) | 23.91 (2.99) | 39.08 (6.55) | -15.16 (-29.32, -1.01) | 0.036 |
| Medicines (SE) | 24.62 (2.91) | 34.39 (5.07) | -9.77 (-21.27, 1.73) | 0.096 |
| Total Health Service Utilisation Costs (SE) | 204.15 (19.56) | 288.27 (50.85) | -84.12 (-191.32, 23.07) | 0.124 |
| **Total Health System Costs** | | | | |
| Total Health System Costs (SE) | 269.81 (19.53) | 288.27 (50.85) | -18.47- (-125.64, 88.71) | 0.735 |
| **Productivity Costs** |  |  |  |  |
| Time costs to service users and families (SE) | 164.70 (12.89) | 154.89 (12.77) | 9.81 (-25.83, 45.46) | 0.589 |
| Productivity losses (SE) | 344.95 (24.85) | 491.22 (26.80) | -146.28 (-218.08, -74.47) | 0.000 |
| **Total Societal Costs** |  |  |  |  |
| Societal perspective (SE) | 779.46 (40.84) | 934.39 (64.81) | -154.93 (-305.51, -4.35) | 0.044 |
| **QALYs** | | | | |
| QALYs gained (SE) | 0.848 (0.005) | 0.837 (0.004) | 0.011 (0.006, -0.002) | 0.092 |
