# Supplementary material for: Sustained effectiveness and cost-effectiveness of the Healthy Activity Programme, a brief psychological treatment for depression delivered by lay counsellors in primary care: 12-month follow-up of a randomised controlled trial
Source: PLoS Med. 2017 Sep 12;14(9):e1002385. doi: 10.1371/journal.pmed.1002385 (PMC5595303; doi:10.1371/journal.pmed.1002385)
Supplement: S2 Text — (PDF) [file pmed.1002385.s014.pdf]

Healthy Activity Program

2013

Authors

Arpita Anand

Neerja Chowdhary

Sona Dimidjian

Vikram Patel

Acknowledgments

The authors would like to acknowledge the following:

- Steve Hollon and Janardhan Reddy for their valuable inputs which helped to enhance the content of this manual
- The investigators group, including Betty Kirkwood, Chris Fairburn, Michael King, Atif Rahman, Ricardo Araya, Pratima Murthy, Helena Verdeli, Terry Wilson, Mark Jordans, Richard Velleman and Vivek Benegal for their involvement throughout the treatment development of the Healthy Activity Program
- Medha Upadhye, Akila Sadik Bepari, Rajashri Deshpande and Mallika Patankar for their tireless efforts in providing the clinical data to inform this manual.
- Sachin Shinde, Madhumita Balaji and Benedict Weobong for coordinating the research work which helped us to contextualise this manual to the cultural setting.
- The health counsellors at the frontline of implementing the Healthy Activity Program and in doing so helping us to fine-tune its contents.
- The General Practitioners, Parivartan (Satara) and Directorate of Health Services, Goa for allowing us access to their facilities to conduct the clinical and research activities which informed this manual.
- The doctors, nurses and other staff of the PHCs and general practices who welcomed us into their clinics and supported us in our clinical and research activities.
- The participants in the treatment development workshops, case series and pilot studies without whom this manual would not have come to being.
- Dielle D'Souza for help in formatting and technical assistance.
- The PREMIUM administrative team who worked silently in the background supporting and encouraging us in developing this manual.

The work that has led to this manual has been entirely funded by the Wellcome Trust through a Senior Research Fellowship grant to Vikram Patel.

TABLE OF CONTENTS		
**Sr No**	**Chapter**	**Page No**
1	Understanding Depression	6
2	Healthy Activity Program	12
3	Style of a Healthy Activity Program Counsellor	15
4	Phase-wise Guide	19
A	Phase 1: Getting started	20
B	Phase 2: Learning together, getting active and solving problems	31
C	Phase 3: Ending well	51
5	Useful Strategies for Specific Problems	55
6	Telephone Counselling	67
7	Role of Medication	70
8	Glossary of contents	73

Introduction

The Healthy Activity Program manual aims at providing counsellors like you with information about counselling patients with moderate to severe Depression in primary care settings. It is designed to accompany the Counselling Relationship manual.

This manual has been developed by the PREMIUM team as part of a five-year project that seeks to develop and evaluate culturally appropriate psychological treatments for two priority mental health conditions –Depression and Harmful/Dependent Drinking. Our research has demonstrated that the type of counselling treatment that is most likely to be effective in our setting is one that uses Behavioral Activation (BA). BA is therefore the treatment that forms the core of the Healthy Activity Program.

The manual has seven separate chapters. We have emphasized a number of salient points within the text and key concepts are illustrated with the use of case examples and scripts. We provide a step-by-step guide for each phase of the treatment and include homework charts and handouts that you can use with the patients. As a counsellor, you may face significant challenges during the course of your work and we have recommended solutions that can be implemented to overcome these.

This manual is meant to act as a practical guide that you can refer to on an ongoing basis to develop and strengthen your skills in helping patients with moderate/severe Depression.

Chapter 1

Understanding Depression

Learning Objectives

In this chapter, we will learn:

- What is Depression?
- What are the symptoms of Depression?
- How to screen for symptoms of Depression

CONTENT

Kamala is a 50-year-old woman who has three children. Her husband left her when the children were small and she has managed to look after them over the years. Her eldest daughter recently got a job and is earning a good income. The other two children are still studying in school. She was very happy that her daughter could share the financial burden of caring for the family but then her daughter got involved with a married man from the locality. She now is living with him and has cut off relationship with Kamala and the two younger children. Kamala has been very upset since this time. She feels very hopeless, tired, and irritable, and she has been having difficulty sleeping and eating.

Manisha is 35 years old, living with her husband who has not had a regular job for many years. He also has a drinking problem and beats her frequently. Her two married sisters support her and her husband and their two children financially. She recently took up a job, but she is unable to concentrate on her work, feels tired, and sleeps poorly. She reports having lost interest in life and attempted suicide two months back.

Harish is a 25-year-old driver. He recently left his job in the city to come back to his village. His wife suspected that he was having affairs with other women in the city and wanted him to come back. Harish does not have a job in the village. He spends most of the time in his house, and he frequently fights with his wife. He reports feeling irritable most of the time, has lost interest in activities that he enjoyed earlier, and feels tired all the time. He complains of disturbed sleep and loss of appetite.

Meena is a 42-year-old woman who feels tired all the time, has generalized body aches, has trouble falling asleep and is unable to concentrate in her work teaching at the local school. Her husband died suddenly in an accident three months ago. She misses him very much, thinks about all the plans they had made for the future, and worries about how she will manage without him. She avoids her friends and family and is absent from work frequently. She spends most of her time alone at home, crying or lying in bed for hours.

We may meet patients like Kamala, Manisha, Harish or Meena during our work as counsellors in the Primary Health Clinic (PHC). With what are they struggling? How can we help them? This section of the manual will help us find answers to these two questions.

WHAT IS DEPRESSION?

It is important to understand the nature of Depression. Such an understanding will guide us both in helping people understand what they are experiencing and helping people out of Depression. Most people are confused by the experience of Depression and have many incorrect ideas.

These are a few important facts to know about Depression in order to address common misconceptions.

- Depression is a stress related mental illness, which causes disturbances in a person’s mood
- Depression affects about 5-10% of the population at any given point of time
- Depression is 2-3 times more common in women than men
- Depression can affect a person’s physical health by increasing the risk of heart diseases and diabetes. When people with physical illness have Depression, they are less likely to seek help for their physical problems and are less likely to follow the recommended treatments.
- Depression, if untreated, can also be fatal. Approximately 1 in 10 people with Depression die by suicide.

Depression **may have many names**

Throughout this manual, we use the term "Depression" but it is important to be sensitive to the way that individuals describe their experiences. Often, it is common for people to describe their experiences as "tension" or with terms like "stress" or "difficulty." Thus, when you see the word "Depression" in this manual, please remember that you are encouraged to substitute for this term the specific language that helps you connect most effectively with your patient. For example, Kamala and Manisha refer to their experiences as 'money problems' and Meena refers to her problems as 'pains'.

Table 1: Misconceptions and facts associated with Depression

**Misconceptions**	**Facts**
Depression is a sign of weakness or laziness	Weakness and tiredness are symptoms of Depression. Depression is an illness, not a character problem.
Depression is caused by possession by spirit forces.	Depression is a condition that is often related to stress in one's life.
Depression is untreatable.	Depression is highly treatable. Both medicine and counselling are good treatment options.
Depression is caused by having a negative (or pessimistic) outlook.	A negative outlook is a symptom of Depression and is not the cause of Depression.
Depression is always hereditary.	Depression may sometimes be related to hereditary factors but it has different components - biological, psychological and social (or stress-related).
The experiences associated with Depression are a natural consequence of the difficulties of everyday life.	Depression is often related to difficulties in daily life, but it is an illness that can result from an interaction of biological, psychological and social (stress-related) factors
Depression is very rare.	Depression affects 5-10% of the population at any given time.
Depression is 'madness.'	Depression is a stress related condition that is treatable.
Depression can only be treated by medication.	Depression can be treated very effectively with counseling.

WHAT ARE THE SYMPTOMS OF DEPRESSION?

The experience of Depression contains multiple components. We can think of these as falling into four broad categories—physical, feelings, thoughts, and actions, as shown in *Figure 2*.

In a primary health clinic (PHC), patients are more likely to seek treatment for the physical symptoms that often go along with Depression — experiences such as tiredness, weakness, aches and pains, disturbed sleep, and poor appetite. When we enquire further, these patients often will have report experiences in the other categories as well. For example, they may report feeling sad or guilty, thinking that nothing will improve for them, or withdrawing from friends and family.

All of us at different times in our lives may have experienced these types of symptoms. At some time or the other, many people have felt tired or sad, had trouble sleeping, had pessimistic thoughts, or were a little withdrawn. But, those symptoms do not by themselves mean that

someone is depressed. It is important to pay attention to three other things as we think about the symptoms of Depression.
First, pay attention to how many and which symptoms are present. A patient may be experiencing Depression if he or she is experiencing many symptoms (listed in *Figure 2*) that include either sadness or loss of interest in daily activities. The more the symptoms, the more likely it is that the patient has a Depression.
Second, pay attention to time. How often are these symptoms occurring and how long have they lasted? If they occur nearly every day for at least two weeks, they may be part of Depression.
Third, pay attention to the effect. Are these symptoms affecting one's functioning — things like how we study, work, interact with our loved ones and handle responsibilities in general? If these symptoms are getting in the way of functioning on an everyday basis, they may indicate Depression.

Thus, the number and duration of the symptoms and their effect on the patient's ning differentiate Depression from everyday sadness.

**Physical**	**Feeling**	**Thoughts**	**Actions**
**Tiredness<br>Weakness<br>Aches and pains<br>Disturbed sleep<br>Poor appetite**	**Sadness<br>Irritability<br>Losing interest in things<br>Guilt**	**Hopelessness<br>Difficulty in making decisions<br>Thinking of oneself as worthless<br>Thoughts about dying or killing oneself**	**Withdrawal from social interaction<br>Inability to work or perform other responsibilities<br>Withdrawal from enjoyable activities**

It is also important to know that Depression tends to occur in combination with other types of problems.
A patient with Depression also may have:

- Symptoms of anxiety — such as heart beating fast, feeling suffocated, chest pain, dizziness, trembling, bodily tension and sensations of pins and needles all over the body. The patient may also report worrying all the time, thinking that something bad will happen and avoiding situations that make them feel anxious.
- Harmful drinking behaviour – sometimes patients may drink alcohol to cope with their distressing thoughts and feelings, which itself can result in further health problems (Refer to Counselling for Alcohol Problems manual for more information on how to help such patients).
- Long-standing or serious physical health problems such as diabetes and heart conditions.

HOW IS DEPRESSION DETECTED?
We use a short questionnaire, called the Primary Health Questionnaire 9 (PHQ 9) **-** to detect Depression. The PHQ 9 helps us measure the symptoms of Depression in each patient and decide whether the patient has Depression currently. PHQ 9 is a paper pencil questionnaire that has items that correspond to the symptoms, above (*Table 2*).

Table 2:PHQ 9 items related to Depression symptom categories

PHQ 9 item	Symptom category
Little interest or pleasure in doing things	Action
Feeling down, depressed or hopeless	Feeling
Trouble falling/staying asleep, sleeping too much	Physical
Feeling tired or having little energy	Physical
Poor appetite or overeating	Physical
Feeling bad about yourself, or that you are a failure, or have let yourself or your family down	Thought
Trouble concentrating on things	Thought
Moving or speaking too slowly that other people may have noticed (or being fidgety or restless)	Action
Thoughts that you would be better off dead or of hurting yourself in some way	Thought

Each item is rated between 0 to 3, depending upon frequency of the symptom in the last 2 weeks. We count one point for each item checked several days, two points for checked items more than half the days, three points for items checked nearly every day. The total score is then calculated by adding all the item scores. Possible scores ranging from 0 to 27, with higher scores indicating greater Depression severity. These are interpreted as follows (*Table 3*):

Table 3: Interpretation of PHQ 9 scores

PHQ 9 Score	What it means
</=10	Minimal depressive symptoms
10-14	Mild depressive symptoms
15 - 19	Moderate depressive symptoms
>/=20	Severe depressive symptoms

TREATMENTS AVAILABLE FOR DEPRESSION

There are different ways to treat Depression. Two broad categories of treatment are talking treatments and medication.

<u>Healthy Activity Program</u>

In HAP, help is offered by talking to patients about their concerns and improving Depression by helping people make changes in their behaviour.

<u>Medication</u>

In this form of treatment, medicines are prescribed for Depression (called antidepressants). This form of treatment seeks to improve Depression by changing the chemical balance in the brain, which is disturbed during Depression (Refer to *Chapter 7* for more information).

SUMMARY

- Depression is a stress related illness that can be caused by multiple biological, psychological and social factors.
- Depression can present itself in different ways; in a PHC, patients are more likely to seek treatment for the physical symptoms associated with Depression.
- The diagnosis of Depression depends on the number of symptoms, how long they last, and how much they get in the way of daily functioning.

- A person suffering from Depression may have additional problems such as anxiety, harmful drinking or physical health problems. It is important to treat these problems in addition to treating the Depression.
- Depression can be treated either by medication or counselling or a combination of both.
- HAP is an effective counseling treatment for Depression.

Chapter 2

Healthy Activity Program

Learning Objectives

In this chapter, we will learn:

- What is the Healthy Activity Program?
- What are the phases of the Healthy Activity Program?

CONTENT

There are different ways to help people overcome Depression. The Healthy Activity Program (HAP) is based on an extremely useful type of counselling treatment for helping people overcome Depression.

WHAT IS THE HEALTHY ACTIVITY PROGRAM (HAP)?

- As we learned in *Chapter 1*, there are four categories of Depression symptoms. HAP focuses on changing the "action" category in order to change the other three categories (i.e., body, feelings, and thoughts).
- The focus of HAP is on what patients are doing (or not doing). HAP treats Depression by helping patients do activities that are pleasurable and activities that solve problems. We may refer to this as 'Doing Therapy'.
- As a HAP counsellor, you will help your patients identify the links between what they do and how they feel, and use this information to identify specific at-home activities that will help your patients begin to feel better and solve problems.HAP is delivered in a maximum of 8 sessions over three phases
- HAP focuses on patients with moderate to severe Depression as detected by the PHQ 9. This is because patients with mild Depression may not need such intensive counselling and will often improve with simple advice and support. In addition, these forms of counselling are as effective as medication in moderate to severe Depression. In fact, counselling is sometimes preferred over medication, for example, in some patients who may not tolerate medication and develop side effects more easily.

HEALTHY ACTIVITY PROGRAM PHASES

The Healthy Activity Program follows a general course over time – these are described as phases of counseling. *Table 4* describes the phases of HAP and identifies relevant sections of this manual.

Table 4: Healthy Activity Progam Phases

Phase	Goals	Description	Manual Sections
Early phase (Delivered in 1-2 sessions)	Engaging and establishing an effective counselling relationship<br>Helping patients understand the Healthy Activity Program model<br>Eliciting commitment for counselling	Includes getting started, understanding the patient's problem using the Health Activity Program model, getting commitment to the treatment and addressing barriers to treatment engagement	*Chapter 3*: Style of a Healthy Activity Program counsellor<br><br>CR manual – Chapter 3
Middle phase(Delivered in 3-6 sessions)	Assessing activation targets and encouraging activation<br>Identifying barriers to activation and learning how to	Includes assessment and activation strategies as well as problem solving when needed.	Chapter 4:<br>*Part 1*: Learning together<br>*Part 2*: Getting active and solving problems

	overcome these Helping patients solve (or cope with) life problems		*Chapter 5*: Additional strategies to overcome barriers
Ending phase (Delivered in 1 session)	Reviewing and strengthening gains that the patient has made during treatment in order to prevent relapse.	Includes summarizing key learnings from the treatment and preparing for situations in the patient's life that may trigger Depression in the future and generating plans to deal with such situations.	*Chapter 4*: Ending well

Moving through Healthy Activity Program Phases

The three phases of HAP provide a helpful guide for the counselor. We move from one phase to the next based on whether the goals of the previous phase have been achieved. The phases may overlap with one another and some patients may need to remain at one phase for a longer time if the goals have not been achieved or if the patient does not show improvement (based on reduction in PHQ 9 scores). The phases of treatment are, therefore, delivered in a flexible manner across 6 to 8 sessions at weekly (preferably)/fortnightly intervals, with each session lasting 30-40 minutes. The first session is always devoted to the goals of Phase 1; although, some patients may require two (or rarely, more) sessions to achieve these goals. For most patients, we would expect to achieve the goals of all phases by session 5 or 6. However, if at the end of 4 sessions, the patient does not show improvement, this means we may have to extend the middle phase. In such situations, we need to discuss this with our supervisors and offer up to 2 more sessions to achieve the goals of the middle phase.

If the patient has not recovered at the end of 8 sessions, the needs of the patient and the progress must be reviewed with the supervisor and the patient can referred to a psychiatrist appointed by the program for antidepressant medication.

Ensuring Treatment Quality

A rating scale, called Q-HAP, is used to assess treatment quality in the group supervision sessions. In each session, the scale is rated by an expert supervisor, a peer supervisor and peers.

SUMMARY

- The Healthy Activity Program is a structured counselling treatment with focus on action
- The Healthy Activity Program may be referred to as 'Doing Therapy'
- The Healthy Activity Program is delivered in phases with each phase having its own goals
- The treatment quality is assessed using a quality assessment tool.

Chapter 3

Style Of A Healthy Activity Program Counsellor

Learning Objectives

In this chapter, we will learn:

- What are the key aspects of the counsellor’s style when delivering the Healthy Activity Program?

CONTENT

All the skills we learnt on general counselling skills in the CR manual (Chapter 2) are applicable when counselling patients with Depression using the Healthy Activity Program. You are strongly recommended to read the CR manual before proceeding as many of the terms used here are explained in that manual. There are some key aspects of the counselling style that are particularly important in Healthy Activity Program and these are highlighted below:

Maintan session structure
Keep the focus on action
Learn together collaboratively
Be non-judgmental
Acknowledge the patient's experience
Be encouraging
Express warmth and be genuine

1. Maintain session structure

As we have discussed, a counselling session is characteristically different to a friendly chat. One of the most distinguishing features is to set and maintain a structure throughout the session. This is extremely important while working with patients with Depression, who can feel easily overwhelmed and discouraged. The components that make up the session are described in *Chapter 4*.

At the start of each session, it is recommended that you and the patient set an agenda, or a plan for the session, together. A clear agenda for each session will help you and the patient focus on what is most important for the patient to make progress. This plan or agenda includes a list of topics to discuss or tasks to complete in the session and provides a guide for you to follow for the remainder of the session.

In general, it is important to remember that setting and following the agenda for the session is:

- Collaborative, such that the session focuses on what matters most to the patient
- Useful, such that patients and counsellors have a guide for conducting the session and ensuring that they stay on track with the goals for the session
- Flexible, such that new topics or tasks can be added, but are best if done so in a clear and collaborative manner by directly discussing and deciding whether to modify the original agenda.

2. Keep the focus on action

Healthy Activity Program emphasizes action and doing things differently in order to help the patient feel better. This is described in *Chapter 4*. This is another way that a counseling session differs from a friendly chat. In a HAP session, we keep our focus on action and limit the time

spent on topics that do are not central to the patient taking action in order to solve problems and feel better. Of course, there are times that it may be necessary to talk about topics that are less central (e.g., when greeting the patient); however, we emphasize discussions that help the patient take action and solve problems.

3. Learn together collaboratively

Healthy Activity Program emphasizes the importance of counselors and patients working together as a team. We can do this by introducing the patient to each step and ensuring that the patient understands the reasons for the steps that we are recommending. We also encourage the patient to take an active role during the session and share responsibility for setting and following the session agenda. We also emphasize working collaboratively when identifying activities that will be targets for change as well as in planning the best way to accomplish these activities.

Often at the start of treatment (or with patients experiencing severe Depression symptoms at any point), it is important for us to take a very active role in the sessions. This may mean that we are more directive in setting the agenda and suggesting actions to take (or even requiring them if the patient is at risk of self harm or suicide), but even at such times, we maintain collaboration by explaining to patients the reasons for what we are recommending.

In addition to collaborating with the patient, it also may be helpful to collaborate with a family member who is close to the patient and may be available to support and help the patient through the treatment.

4. Being non-judgmental

It is can be challenging to work with people with Depression when they do not complete activities or come to sessions or other problems arise. Becoming critical or judgmental of patients at such times can make counseling even harder. Being judgmental often gets in the way of active problem solving because it makes it harder to learn collaboratively with the patient. For example, when patients report spending all afternoon lying in bed instead of doing a homework plan we developed with them, we may think, “This is terrible!” or “she is making a mess of things!” Practicing a non-judgmental style, instead, helps us to focus specifically on what happened, rather than on our opinions or reactions. In this example, we might ask in a direct and matter of fact way, “what was happening when you got into bed? What were you feeling or thinking then? What happened as you were lying in bed?” Being non-judgmental helps us to shift to learning collaboratively together.

Also, often patients have been subjected to judgmental comments from others like ‘get yourself together’ or 'you need to be strong.’ They also may have many judgmental thoughts about themselves, such as “I’m a bad person.” If we are judgmental, it can make it harder for patients to connect or work with us in sessions. Our non-judgmental attitudes can help us build trusting relationships with patients and support the patient in opening up and sharing more with us.

5. Acknowledging the patient’s experience

In addition to being non-judgmental, it is also important that we acknowledge the patients’ experience and communicate that their experience makes sense to us. For example, we may need to acknowledge that it makes sense that when the patient feels depressed, she/he withdraws from friends and family, doesn’t complete homework, or fails to attend counselling sessions. Also, many patients with Depression attribute their problems to physical illness and focus a lot of attention on their physical health. It is important to accept and acknowledge the patient’s experience of such physical health concerns. You can build on such acknowledgment by explaining the link between physical health and stress and explaining how the Healthy Activity Program will help them feel better.

6. Encouraging progress

A counsellor’s role is to encourage the patient every step of the way, looking out for even the smallest signs of progress and improvement. The nature of Depression is such that it tends to take away from the patient’s confidence and motivation. It is our job to help patients set small manageable tasks and encourage even the smallest signs of action and improvement in mood.

7. Express warmth and be genuine

Being warm and genuine with clients also can help to establish a positive working relationship. We can communicate warmth verbally and non-verbally in ways that are genuine and natural for each of us. Verbal expressions of care (e.g., “I’m happy to see you today” or “I’m sorry that was so hard”) or non-verbal expressions of warmth (e.g., a smile, body language, eye contact, tone of voice) are simple ways in which this can be done.

SUMMARY

- The Healthy Activity Program counsellor maintains a specific style throughout the counselling treatment.
- Key aspects of this style include setting and following an agenda, keeping the focus of counselling on action, and learning together with the patient.
- It also is important for the counsellor to be non judgmental, acknowledge the patient’s experience, be encouraging, and express warmth and genuineness.

Chapter 4

Phase-wise Guide

Learning Objectives

In this chapter, we will learn:

- How to deliver each session of the Healthy Activity Program in a step-by-step manner

PHASE 1: GETTING STARTED

Phase 1 is delivered in 1-2 sessions

Anjana is a 35-year-old married woman with 2 children, aged 14 and 10. Her husband has a drinking problem and has not kept a regular job for several years. Anjana has had the responsibility of supporting the family and works as domestic help. In addition to her work, she also takes care of her children and home singlehandedly. Anjana presented to the clinic with complaints of tiredness, backaches, and headaches. She describes feeling helpless to change the problems in her life, and she reports feeling sad and hopeless about the future. She does not know how to cope with the burden of caring for her children along with increasing financial pressures in the family, and she is distressed by the frequent conflict with her husband. Her health problems also are making it hard for her to keep up with her job, which adds to her concerns. She stays in bed as much as possible when at home, and she avoids neighbours as she does not want to talk about her problems. Her sleep is disturbed and she has been eating poorly. Anjana is experiencing Depression, which she describes as 'tension'.

Goals of Phase 1:

- Engaging and establishing an effective relationship
- Helping patients understand the Healthy Activity Program
- Eliciting commitment for counselling

Outline for Phase 1:

- Introducing ourselves
- Establishing agreement about the length of your first meeting
- Setting an agenda
- Getting to know your patient
- Assessing suicide risk
- Explaining the Healthy Activity Program and providing hope and encouragement
- Talking about the specifics of counselling
- Involving a SO
- Eliciting commitment
- Overcoming barriers
- Addressing the patient's chief concern e.g. referral to social agency, advice for sleep problems
- Planning homework
- Summarising
- Setting next session date
- Completing documentation

Outline for Abbreviated Session:

- Introducing ourselves
- Establishing agreement about the length of your first meeting
- Providing a brief description of the counselling (refer to the patient brochure) with an emphasis on providing encouragement and hope
- Assessing suicide risk (based on PHQ 9 score)
- Setting next session date
- Explaining about what we didn't have time to talk about - this will include discussing of the patient's problems, their effect on the patient's health and specific aspects of the counselling. Reassure the patient that this will be covered in the next meeting.

- Providing the patient booklet and encouraging the patient to read the first page before the next session. If patients can't read, they can ask a SO to read and explain the information to them.
- Completing documentation

Introducing ourselves

Getting started is a collaborative process between us and the patient. The patient is asked to share information about his/her life, challenges, goals, and values. We share information about Depression and how to overcome Depression using HAP, and are asked to make connections to the patient's life and experiences. Thus, getting started is a conversation, rather than a lecture.

At the start of HAP, it is important to build a relationship with the patient. The content of the first session is critical, and using all of the elements of the HAP style is critical also. Pacing the session so that the patient understands the information and believes that we have understood their experience is important.

Since we are providing counselling as part of the program, we will be referred patients whom the Health Assistant has detected to have a moderate or severe Depression using the PHQ 9 (i.e. scoring 15 or above). Thus, we will need to prepare ourselves for the session before we meet with the patient. We then greet the patient and explain about confidentiality. These important steps are detailed in the CR manual, Chapter 3.

As patients attend the clinic primarily with physical complaints, we may begin by asking the patient what brings him/her to the clinic.

'Anjana would you like to share what brings you to the clinic today?'

Once the patient shares the presenting complaint, we will reinforce that the doctor in the clinic will address or has already addressed the physical health problem. In addition to these complaints, we will also explain to the patient that the scores reflect that he/she might be having some stress related emotional difficulties as well. We will then state that as the physical complaints are being addressed by the doctor, if the patient permits, we could address the stress related emotional problems.

'So the doctor has/will help(ed) you address your pain and fatigue. We have also noted that you are having some other difficulties which are related to stress and tension. I would like to discuss these with you in detail to see how we can help you. Would you like to talk about that together now?'

Establishing agreement about the length of the first session

Most patients will not have planned to do a counselling session as part of their health clinic visit, and some may not be able to stay for the entire duration of 30-40 minutes required of a first HAP session. It is important to check with the patient about their availability and make a collaborative plan for how to proceed:

"This discussion will take about half an hour to 40 minutes. Can you stay for this today?"

If the patient expresses agreement, then we proceed with the full first session outline by setting an agenda.

If the patient expresses inability to wait for the entire duration of the session, we follow the brief session outline. We may say:

"Then I would like to take about 10 minutes to describe to you how I can help you with your problem; is that ok?"

Setting an agenda

In the first session, it is important to orient the patient to the process of agenda setting as the starting point for the session. We begin by making a list of items that we would like to cover in the session such as:

- Asking about the patient’s problem/s
- Explaining counselling and how it can help them
- Making a plan for future sessions
- Setting the next session date

The next step of agenda setting is to ask the patient if he/she is in agreement with the list and if there are any additional items that he/she would like to add.

Many patients will be unfamiliar with a structure that invites them to be active participants with their healthcare providers. Hence, in the first session we take the lead in setting the agenda. In subsequent sessions, however, we first invite the patient to list agenda items to which we then add ours.

Example: *It’s lovely to see you today, Anjana. At the start of each of our meetings, I would like for us to make a plan about what we will talk about together. This will ensure that we focus on what is most important for you and will provide a guide for us to use during the session to make sure that we are staying on track. I have a few things that I would like to speak with you about today. Specifically, I would like to know more about you and the problems you are facing. I would then like to explain to you about the counselling treatment that can help with your problems and obtain your agreement to participate in this. We can then make a plan of how we can proceed with this treatment and arrange our next session together. How does this list sound to you? Is there anything else you would like to discuss?*

<u>Getting to know your patient</u>

The Healthy Activity Model guides our understanding of what Depression and how to treat it. It has four main parts, which are framed as questions that are simple and easy to recall. It provides a useful guide for us when getting to know our patient as well a useful guide for us when explaining the counselling process to our patient. Often, it is helpful to draw the different elements of the model, corresponding to these four questions, as you discuss the questions together. The final drawing looks like this:

Figure 1: Healthy Activity Model of Depression

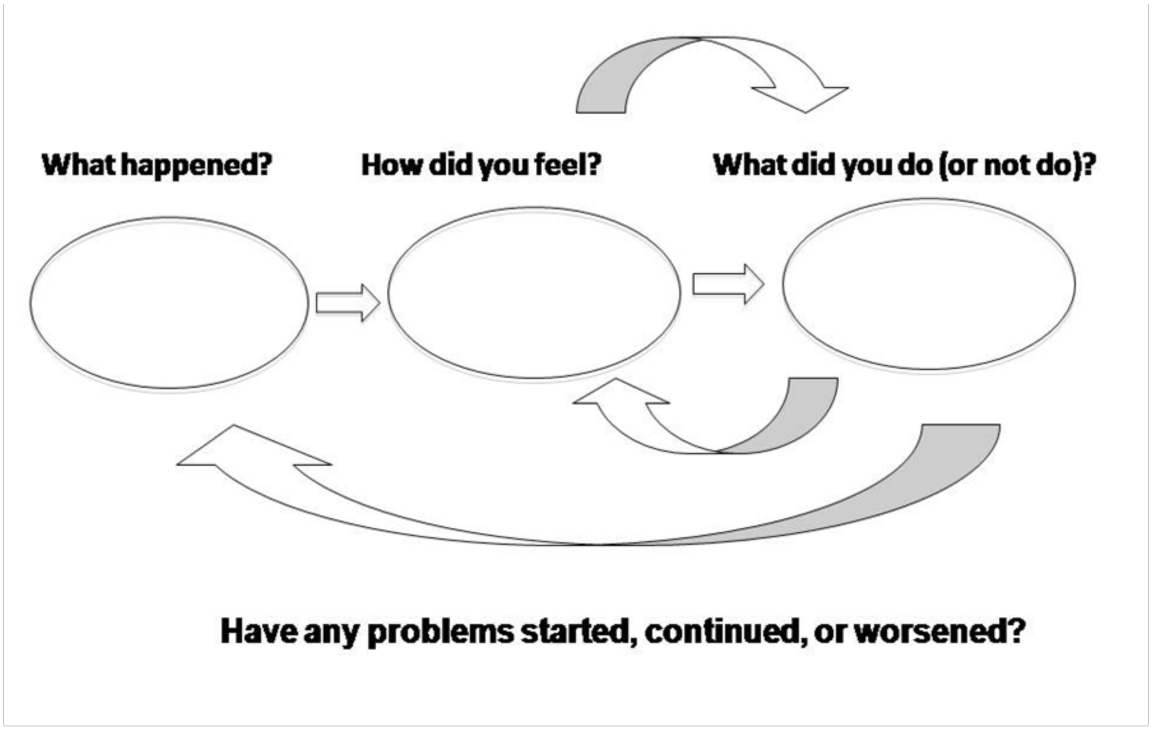

At the stage of 'getting to know your patient' it is important to keep the structure of the session in mind but at the same time ensure that we do not turn the session into a question and answer session. Give your patients time to share thoughts and feelings so that you can understand their experience. Aim for an "unhurried feel" in conducting the session.

The first question that we ask is: "What happened?"

It is important to identify possible factors that either contributed to Depression starting or that are maintaining Depression currently. Often, the most helpful factors to identify are events or stressors in the patient's life; however, biological factors can be helpful to acknowledge too (e.g., for the patient who says "everyone in my family has tension" or the patient whose Depression is linked to biological reproductive transitions such as pregnancy or menopause).

- *In Anjana's case, increasing financial pressures and health problems are important.*

Often, it is helpful to ask say to the patient, "We think about Depression as occurring in response to things that happen in your life. Did anything happen when you started feeling down (or stressed)? Or has anything been going on in your life now that makes your life more stressful or harder (or that keeps you feeling down)?"

If the patient has difficulty identifying such factors, we can offer possibilities of common factors. We might say, "It's common for people who are feeling down/ stressed to have struggles with things such as…" A common list of factors might include:

- Acute stress, defined as a difficult event that has just happened (e.g. death of a loved one, loss of a job, serious accident or injury)
- Long-term stress, defined as problems that the patient has been dealing with for a long time (e.g. work related or relationship problems; lack of safety in one's neighborhood or home; long-term financial problems; or having to care for a sick family member)
- Adverse childhood experience in the past (e.g. abuse, neglect or trauma)

The second question is: "How did you feel?"

It is next important for us to work with the patient to explore how he/she felt in the context of what happened. What was the patient's experience of going through difficult circumstances or what is their experience now? How does he or she feel? In particular, what emotions or sensations in the body are experienced?

- *Anjana feels sad, hopeless and helpless. She also has physical symptoms such as headaches, and backaches.*

We can ask, "when you experience such events (e.g., tension with your husband), how do you feel?" We may explain that experiences that are common in patients under stress are:

- Sadness and/or irritability
- Low interest or motivation
- Decreased energy- feeling tired and fatigued
- Lowered attention and concentration- inability to study or keep at one task for long
- Thoughts of hopelessness, worthlessness and helplessness

If the patient has difficulty in recounting these experiences, we can refer her/him to the items of the PHQ 9 that were scored positive and ask about these experiences

It also is very important to explain that these feelings are normal in the face of life's challenges.

The third question is: "What did you do?"

It is important to identify with the patient how he/she responded behaviorally to the experience of the events and subsequent feelings. This discussion focuses specifically on what the patient does (or often, does *not* do) when feeling down. This is the basis of HAP.

- *The main actions for Anjana are staying in bed, avoiding contact with neighbours, eating poorly, and possibly the ways that she addresses the problems with her husband.*

It is helpful to ask simply and directly, *"When you are feeling stressed (sad, tired, etc.), what do you do (or not do)?"* It often is helpful to explain as well that it is very common for people to start or stop doing certain activities when feeling down. These can include:

- Not meeting or talking to friends and family
- Not going to work
- Doing routine activities e.g. sleeping, eating, hygiene routines in disrupted ways
- Stopping or reducing physical activity (staying in bed, not exercising, etc.)
- Increasing conflict with people close to them
- Thinking again and again about their problems.

If the patient does not easily describe activities, you can ask: *Are there activities you were doing earlier that you have stopped doing since your stress began?*

Again, it is very important to emphasize that it makes sense that one would start or stop doing such activities when feeling all the emotions and sensations that go along with feeling stressed.

<u>The fourth question is: "What is the connection between what you do (or don't do) and how you feel and the problems/stressors in your life?"</u>

It is important to discuss with patients the ways in which their actions (or lack of actions) may be keeping them stuck in Depression.

Many times, the activities patients engage in (or stop engaging in) help them feel better temporarily but over time make things worse. The less one does, the less one feels like doing! We can say

"It makes sense that you want to withdraw when you are feeling down, but the problem with this kind of behaviour is that it keeps you stuck in feeling low/down/stressed as you are less engaged in things that make you happy.

Patterns of withdrawal also can cause the problems in one's life to build and increase. The less one does, the more stressors add up or worsen. We can explain this by saying (for example to Anjana):

"When people face a stressful situation like financial problems and lack of support, they tend to do certain things like you did by not meeting neighbours or staying in bed. This may have helped you for some time, as you did not need to talk about your problems and felt temporarily less tired, but in the long run it has also prevented you from doing things that help you feel better and has left you feeling less supported in coping the problems you are facing. Do you think staying alone at home makes your stress/sadness worse and again this makes you not want to do anything? So this cycle goes on and on? Does this sound similar to your experience?"

It is exactly these activities (or withdrawal from activities) that Healthy Activity Program focuses on. In, Healthy Activity Program, we call these <u>activities</u> that don't make you feel good and our counselling sessions work to decrease such activities.

**Example of activities that don't make you feel good**
Withdrawing from social activities or contacts
Watching TV for hours at a time
Lying in bed for long periods
Not attending work
Neglecting personal appearance and hygiene

Finally, it is useful to ask about things that patients do that help them to cope as well or things that they used to do that were helpful in their lives. In Healthy Activity Program, we call these activities that make you feel good and our counselling sessions work to increase such activities.

**Examples of activities that make you feel good**
Speaking to a friend
Playing with a grandchild
Spending time in hobbies such as sewing
Praying or reading religious or motivational books
Going for a walk

Suicide risk assessment

We need to assess risk of suicide among all patients. If during the course of our assessment, we find that suicide risk is present, or if the suicide item is positive on the PHQ 9 (i.e. item 9), then we must add this to the agenda and this becomes the immediate focus of the session. Assessment and management of suicide risk is detailed in the CR manual (Chapter 4).

Talking about the specifics of counselling

In the first session, we focus on learning about the patient, which requires asking patients many questions. However, it is also important to give the new patient some information about HAP.

Practical information includes:

- Our training and by whom we are supervised
- How long is the counselling – the number of sessions and over how much time.
- Where the sessions will be conducted –at home, in the clinic or over the telephone
- How we can be contacted outside the sessions, and under what circumstances (e.g. only in an emergency).

"As I mentioned, I'm working as a counsellor in this health centre. I've received training in providing counselling for patients with stress related problems. I work with supervisors, who have been trained by experts in this field. They will supervise my work.
As we have been discussing, I would like to once again share with you that the doctor in the clinic will be providing you any medicines that you need. As far as the counselling is concerned, in our experience, we find that we need about 6-8 sessions for about 30-40 minutes each time, spread over 2-3 months or so, to help you feel better/overcome your difficulties.
Ideally, our sessions will take place at your home. We will come to your home at a time that is convenient for you and conduct the sessions. You do not need to travel to the PHC. If, however, you prefer to come to the PHC, we can arrange a suitable time for that too. Another option is for the sessions to be done over the phone in case home visits or PHC visits are not possible. It is important that we have regular sessions and complete the treatment, so these options are available to you. In addition to our meetings, we will plan some homework that you can practice between sessions. In this treatment, we will be working together as a team. Do you think we can join hands to deal with these issues better? In case you need to contact me in between sessions, you may call me on XXXX number. I am available between 9am to 5pm from Monday to Saturday"

Remember to always ask the patient if they have any questions about the treatment and respond to these as best as you can. Examples of common questions are listed below.
Finally, it is often helpful to provide encouragement during the first sessions. It may be useful to ask to ask the patient how life would be different if their stress or tension was addressed. Based on the response, we provide encouragement and hope that through the process of counselling, they can be treated.

"Anjana, we have talked about the problems you are facing. Could you tell me how life would be if you were able to manage these problems better? (patient responds and the counsellor explains further) *Through counselling, we can work together to break this cycle of* Depression *(or tension or stress, using the patient's term). We can plan steps for you to do things to feel better and change stressful situations in your life so that you may be able to……"* (summarise the patient's response). *This approach has been useful for many people struggling with similar problems like yours"*

Table 5: FAQs about the specifics of counselling

FAQs	Answers
Are medicines not enough?	Counselling is known to increase the chances for recovery. In fact, the benefits of counselling can last even after the sessions are completed. If you are receiving medicines from the doctor for your other health problems, you can continue to receive these while you do the counselling.
What if I cannot come to the clinic to see you?	We can have sessions at your home, if this is ok with you. We can also arrange to speak on the phone if you are unable to come to the clinic. Let me know which option you prefer.
Can you speak to my husband/wife/sister/mother to solve my dispute with them?	I will be very happy to meet your relative so he/she can understand the nature of your problem and what they may be able to do to help. Please ask them to read this handout and bring them with you when we meet next. It will be very useful to have them involved in your treatment.
Can this counselling really help me? I am already doing so many activities but I still feel stressed.	We will be working together to examine these activities and see how we can use them to help you feel better. We will also be discussing ways to deal with the problems in your life. This counselling has helped many people with problems such as yours. Let us try it and see the results.

Involving a Significant Other (SO)
People suffering from Depression often don't understand what they are experiencing or what to do that will help them feel better. Unlike physical illnesses that are often visible, mental health problems are often hidden and not easily recognized. The signs and symptoms of Depression may be seen as a character problem (e.g., laziness or weakness), and people with Depression may have been told that they are to blame for their struggles. As a result, people with Depression may not talk about their experiences openly and may wonder why they cannot make themselves feel better. Given this context, an important part of counseling involves helping family members understand what Depression is and ways to treat it.
The guidelines for involving a significant other are given in the CR manual (Chapter 5).
Some important things to remember:

- Ask the patient if they would like the SO (who has accompanied them to the clinic) to be present for a part or the whole of the session. Some patients may be comfortable if the SO attends the entire session but others may want to describe their problems to you without the SO being present and only have the SO join in when you are explaining the Healthy Activity Program or the specifics of counselling.
- Discuss with the patient the possible benefits of involving the SO in counselling.

- Although it is useful to involve a SO, it is not essential. For instance if the patient is showing commitment to the treatment and does not wish for someone else to be involved, this need must be respected and the patient should be encouraged for their efforts.
- If the SO attends the session, then before you end, ask the person if he/she wants to provide any further information about the patient's problem/s and if he/she has any questions.
- Encourage the SO to read the handout and return with any questions he/she may have. The handout can also be used by the patient to explain to a SO about the counselling that he/she will be receiving in the clinic.

Eliciting commitment

It is important to confirm the patient has understood what counselling entails and is willing to proceed with the treatment. Participation in counselling is a choice that the patient makes, so eliciting commitment is an important focus of the first session. We need to give patient some time to talk about their understanding, clarify their doubts, and go over parts that they may not have understood.

"Thank you for coming to the session today. I hope it was helpful to you; I think we have had a very good discussion about the problems you are facing, in terms of what it is exactly, how you got it and what you can do about it. I am very keen to know what you think about the treatment. Would you briefly explain what you have understood from our discussion and how willing you are to participate in this counselling?"

Address barriers

It is important for us to explore barriers that may interfere with patient's engagement in the treatment. Some patients may also refuse treatment, in which case we might explore their reasons for doing that in a matter of fact way. We could say:

"I'm curious about your refusal to attend counselling, could you help me understand it better."

Apart from patient-level barriers, there may be barriers at the PHC level or counsellor level. The table below provides a list of commonly encountered barriers with suggestions of how to address these.

Table 6: Solutions to overcome common barriers commitment to treatment

	**Barriers**	**Solutions**
Patient level	Cannot attend counselling sessions due to practical barriers such as<br>Transport cost<br>Lack of time – e.g. daily wage earners who cannot attend due to loss of wages if absent from work<br>Care giving responsibilities<br>Elderly who cannot travel alone and need to be accompanied to the clinic	Home visits are offered as the primary option. If the patient is not comfortable with home visits then telephone counselling is suggested. Flexibility in scheduling sessions e.g. conduct sessions once in two weeks instead of weekly sessions<br>Patients who are care providers may be helped by actively looking for people who can provide care in their absence. We may discuss with the patient how to enlist help.

	A SO, usually the patient's husband, does not allow counselling	If the SO has accompanied the patient to the clinic, invite him in and engage him in the counselling process.<br>If he is absent, encourage the patient to share the handout with the SO and invite the person to participate in a session.
	Patient has no time to wait for first session	Time of session to be shortened – provide brief session - with emphasis on engagement and addressing barriers to follow-up
	Patient has significant social stressors such financial problems and does not see how 'talking' will help	Explain that you can help the patient address social stressors and do so as soon as possible through referral for appropriate services or problem solving in session
	Patient preoccupied about physical health and attributes problems exclusively to physical illness	Physical illness beliefs to be addressed and explain the mind-body link. Emphasise the role of physical symptoms in depression.
	Patient with depression expresses preference for medication rather than counselling	Counsellor explains that counselling is an option in ADDITION to any medical treatment, not INSTEAD of, and that this addition can further improve the chances of long-term recovery.
	Patient is uncommunicative and doesn't open up about his/her problems	Spend greater amount of time in engaging the patient and explaining how the treatment will help.<br>Assure the patient that all information will be confidential.<br>If the patient has come with a SO you may ask the person to wait outside while you speak to the patient alone.

	Patient is suffering a personal crisis, for example, recent loss of a loved one or patient is emotionally distressed and crying during the session	Allow time for the patient to express their grief or distress. Listen and respond with in warm and genuine ways. Follow the steps in dealing with personal crisis (CR manual, chapter 4), and postpone the other tasks of session 1 for subsequent sessions.
PHC level	Counsellor not seen as part of PHC team	Engage the doctor and other PHC staff<br>to encourage patient to receive counselling
Counsellor level	Failure to engage patient	Remember that the first session is a conversation. It is important to allow time for listening to the patient's experience. It also is important to share your knowledge so that the patient has access to accurate information about counselling.
		Provide patient material<br>Actively involve the SO and elicit their help in ensuring the patient attends counselling session.

Addressing the patient's chief concern/s

In order to effectively engage the patient in counselling, it is important to address her/his main concerns. For example, if the patient has social problems for which there are already existing welfare schemes, we can discuss with the patient how she/he can access these. See CR manual Chapter 6 for guidelines on how to do this. For some patients with depression, their sleep problems are very distressing; often, we can help with sleep problems by providing them with simple tips (refer to *Chapter 5* of this manual for further details). There may be some problems/concerns that the patient has that will require more detailed problem solving. If we think these problems require urgent attention (for example, getting help for a serious medical condition), we can begin the steps of problem solving in this session. Otherwise, we can reassure the patient that we will help them address this problem in future sessions. This reassurance is important so that patients know that we are listening to their concerns and will help them deal with these.

Planning homework

In Healthy Activity Program, patients are encouraged to do work between sessions, whether it is monitoring their activities or practicing a skill. These are the steps involved in planning the homework at the end of the first session:

Explain the homework: At the end of the first session, the patient is given the booklet and the handout containing information for the significant other. We explain the use of the booklet to the patient by saying:

"This booklet contains information about methods to deal with your problems and some blank charts that we will be using during our counselling sessions. Before we meet for our next session, can you read the first sheet? It will help you understand the nature of your problem and what we are doing here in these sessions.

If there are any questions you have about the information, I will be happy to answer these when we meet Please bring this booklet when you come for the next session (if the next session is planned in the PHC)".

If the patient is illiterate, say:

"Can you ask someone in your family (or a friend) to read this to you"? In addition, we give the patient the handout for the significant other, saying: *"This leaflet will help your close family/friends understand your problem better. Please give it to them to read and encourage them to come for the next session with you. I can then answer any questions/concerns they may have".*

Ask for barriers in performing the homework and address these: *Will you be able to do the homework? What may help you do this before we meet/speak again?*

Remind**:** *I hope that the next time we meet you will tell me what you have understood about counselling.*

Summarising

Summarizing refers to providing a brief description of the session. The summary should include the key points that were discussed and the homework for the coming week. A good summary is short but comprehensive so that the patient remembers the key points that happened in a session. It gives the patient an opportunity to summarize the information that was provided so that we can understand how effectively we have communicated the information and whether we need to correct any doubts/misconceptions that the patient or that we may have.

We can say:

"Please tell me what we have discussed in this session."

After the patient says what he/she has learned, repeat his/her ideas and add these things, if necessary:

In this session:

- *We have understood the effect that your stress has on your health*
- *We know that due to your stress you often find it difficult to do activities you were doing earlier and this makes you feel worse.*
- *We have agreed to meet/speak regularly at your home/in the clinic in order to discuss ways to deal with the stress and do activities that will help you feel better*

Setting next session date

The time and place for the next session is best arranged collaboratively with the patient.

As far as possible we try and arrange it after 7 days at the patient's home but must remember to be flexible. For example, it may be more convenient for the patient to see us when he/she has the next appointment with the doctor in the clinic. Sometimes patients may not return for up to a month. While this is not ideal, we may need to suit the patient's convenience. We can try and arrange telephone sessions in between the home visits and clinic visits even if they are brief check-ins with the patient. Please remember to note all contacts in the summary of contacts.

We enter the next session date in our phone diary along with a reminder to call the patient prior to the session. (Please refer to the CR manual)

If the patient is not keen for a home visit, encourage him/her to come to the PHC. Ask if the patient is scheduled to come to the clinic for a follow up with the doctor and fix an appointment to suit this timing. If neither is possible, confirm that you will conduct a telephone session. You can say:

"Are you likely to come to the PHC to meet with your doctor? We can fix a time for a session on the same day. We can also arrange a suitable time/day to speak on the phone and I can call you on the number that you provide. Let me know which option you would prefer".

"If you need to contact me …" (we provide details and follow guidelines as in CR manual)

Completing documentation

After the patient leaves, we need to complete the documentation. The importance of doing this is described in the CR manual, chapter 6. The documents we complete at the end of this session are:

- Clinical record form
- Daily register

PHASE 2

Phase 2 is delivered in 3-6 sessions and has two parts:

Part 1: Learning Together
Part 2: Getting active and Solving problems

Part 1: Learning together

Goals:

- Identifying activation targets

Outline:

- Reviewing progress
- Setting an agenda
- Reviewing homework
- Learning together
- Encouraging activation
- Involving a SO in learning together
- Planning homework (including addressing barriers)
- Summarising
- Setting next session date
- Completing documentation

Introduction

Once we have understood the patient's problem, it is important to select treatment targets by defining the patient's goals and then helping him/her move in the direction of achieving those goals.

Steps

Reviewing progress

Checking progress means measuring how much the patient's symptoms have increased or decreased since the last session. Doing so gives us an objective report of the patient's current state, helps to guide the treatment focus, and allows us to provide feedback to the patient about how his/her condition is progressing.

How is it done?

We check progress by using the PHQ 9. We compare the current PHQ 9 score with that obtained at the previous visit and provide feedback to the patient.

"Based on the questions I have asked you, it seems to me that you are feeling better than you were when we last met. Your earlier score was 19 while today it is 15. Does this fit with how you have been feeling"

Some patients may not be able to understand the significance of the scores. In such cases it is useful to share feedback in words.

"From your answers to these questions, it sounds like you have been feeling a bit better. How does that fit your experience this past week? Or, if there is no significant improvement, you can say, "Based on the feedback you are sharing with me, it seems that you have been feeling more or less the same as the last time we met. Is that correct?" you can then add, "We will focus more on some of these issues today and see how we can help you further."

If the suicide item is positive on the PHQ 9 (i.e. item 9), we need to assess and manage suicide risk as an immediate priority. If item 9 is negative, we may proceed to the next steps in the session.

Setting an agenda

Agenda setting is most useful when it is done collaboratively with the patient. It is often helpful to explain to patients the rationale for using agendas to guide sessions (link to *Phase 1*). It may be necessary to explain a few times the value of agenda setting and to reinforce for patients that we want them to be active collaborators with us. We need to take time to pause in order to allow the patient to contribute items to the agenda or to ask questions.

In order to ensure patient's contribution in the process of agenda setting we can begin as follows:

Go over the list of problems the patient had described in session 1 (i.e. answers to the first question in the Healthy Activity Program) and ask the patient which, if any, of these problems they would like to discuss in the session. This is also important as it provides continuity between sessions.

- Go over any new problems that may have been identified while reviewing progress and ask the patient which, if any, of these they would like to discuss.
- Based on the patient's responses, make a list of agenda items in which we add things that we would like to discuss to the patient's list, such as:
- Asking about the homework the patient (i.e., reading the handout).
- Recording the various activities the patient performs and how they feel when doing these
- Making a plan to identify at least one activity that can help them feel better and scheduling this activity till the next session
- Setting the next session date
- The final step of agenda setting is to ask the patient if he/she is in agreement with the list and if there are any additional items that he/she would like to add.

Example: *It's lovely to see you today, Anjana. As I explained the last time we met, at the start of each of our meetings, I would like for us to make a plan about what we will talk about together. This will ensure that we focus on what is most important for you and will provide a guide for us to use during the session to make sure that we are staying on track...Are there any topics you want to make sure we address together today? (pause for patient response). In the last session, you told me about the problems you are facing with…. Would it be helpful to discuss any of these problems together today?(pause for patient response) That's great. I have a few things to add as well. I would like to review with you the homework we had agreed upon in the last session. I would also like to talk about the activities you do and understand more about these. And, as always, I will want to end with some homework you can do until we meet the next time. How does this list sound to you? Is there anything else you would like to discuss?*

Reviewing homework

Because the Healthy Activity Program is an action-oriented treatment, in each session, we work together to develop an action plan (or homework) for the patient to do prior to the next session. Thus, in each following session, it is important to review the patient's experience with the homework. The review of homework helps us identify next steps for the patient to take.

Ask simply and directly about the patient's experience with the homework assigned in the last session. For example, we may say, *"How did the homework go this week?"* or *"what questions do you have about what you read?"*

Ask the patient to describe their understanding of the Healthy Activity Program. We may say, "*can you tell me what you have understood about the counselling treatment?"* This is an opportunity for us to fill in parts that the patient did not understand or correct any misunderstanding.

What to do if the patient didn't do the homework

It is important to encourage any progress that the patient has made with the homework and to review what was done (or not done) in detail. If the patient has not completed the homework, it is important to ask directly, *"what happened?"* or *"what got in the way?"* It is important to address this topic in a matter of fact and non-judgmental manner. Learning from what got in the way of completing the homework is important in knowing how to help the patient take the next steps.

Although we may feel awkward asking about homework that patients didn't complete, such discussions are essential in counselling. It is not possible to help the patient if we do not know what barriers he/she is facing. It is more common than not for patients to not complete the homework. One way of overcoming this difficulty is to complete the task that was given as homework in the session with the patient. In this way, the responsibility of completion is shared and the patient may feel supported.

The Foundation of Learning Together

The Healthy Activity Program guides patients to do activities that help them feel better, solve problems, and achieve goals.

How do the counselor and patient know what activities will help? They learn together by identifying the links between activity and mood in the context of the patient's daily life.

We need to identify the activities that help the patient feel better and those that do not help the patient feel better in order to build effective action plans. A patient with depression is likely to report more activities that do not help them feel better in their routine. Thus, it is very important to assess a range of activities that the patient is doing and the effect of the activity on the patient's mood. We can then plan with the patient how the activities that help them feel better can be increased by skillfully making them a part of the patient's routine and how activities that do not help them feel better be decreased through problem solving.

We may also use terms like 'things that make you happy' or 'things that make you sad' that convey the same meaning but may be more easily understood.

There are two main ways to identify activities that help the patient feel better and activities that do not help the patient feel better.

First, the Healthy Activity Program model is useful for assessing the links between activity and mood. This is primarily used in discussion during sessions but also can be assigned for the patient to use in between sessions. Often patients will come to sessions with concerns about the period since the previous session, such as arguments with friends or family or times at which they felt particularly down. We can inquire about these situations, asking specifically about the

elements of the Healthy Activity Program: What happened? How did you feel? What did you do or not do? And, what connections were present between what you did and how you felt. We can orient the patient to the use of the Healthy Activity Program model in specific situations by reminding the patient,

"Do you recall how we talked about the program to understand your feeling /stressed/troubled recently? Well, we can use that same approach to understand what happened last week. It will help us identify the links between what you do and how you feel and knowing that will help us develop effective action plans for you to try. Does that sound ok?"

Second, we can use an activity calendar (pg 15 of the patient booklet) to assess activity and mood. It involves recording activities that the patient has been engaged in during the day and mood at the time of performing the activity.

Steps in explaining the activity calendar:

1. It is necessary to explain to the patient the reason why filling the calendar is an important part of the counselling. First we remind them of the HAP model and how the activities they do (or don't do) can affect mood and stress. Then we can say, "*It is helpful for us to have a detailed picture of the different activities you perform during the day and to understand how you feel when you do these activities. We can then choose activities that make you feel better and discuss how you can increase these. Similarly we can discuss how to reduce those activities that make you feel worse. What do you think about this?*
2. Next, we start the calendar with them in the session. You can say, "*We can use this chart to help us understand what activities help you feel better. Is it ok for us to work on this together now? Could you please start by telling me what you did before coming in to the PHC? How did you feel when you were doing that activity? Let's talk about what you did yesterday too... Can you tell me what time you woke up yesterday?"* Pause for reply. *"What did you do after waking up?"* Pause. *"What did you do next?"* and so forth.

Also ask about how the patient felt during these activities. How the patient feels when performing an activity is the key to knowing whether an activity made them feel good or did not make them feel good. Some patients we see do not describe feeling happy or sad but rather describe their mood in physical/somatic terms such as 'tension' or feeling well/unwell. It is important for us to use whatever terms the patient does when explaining mood rating. The different ways to rate mood are:

Table 7: Methods to rate mood

**Mood ladder:**<br>We can ask the patient to rate his/her mood along the rungs of a ladder. So, we may ask the patient:<br>*"If lowest step on the ladder is the worst you have felt and the top step the best you have felt, at which step would you place your current mood?"*
Good / Bad<br>Some of the patients whom we see may not report in detail on the intensity of mood. For such patients, it may be helpful to ask them to choose one of two categories, such as, *"do you feel happy or sad?"* or *"do you feel good or bad?"*
Emoticons<br>For patients who cannot read or write, we can use icons like □□ to identify how they are feeling

Using a tick and a cross<br>We can use a tick mark (√) to indicate a positive mood and a cross (X) to indicate a negative mood.

3. Identify any links between activity and mood with the patient. For example, ask the patient about your observation that she felt better when making dinner than when she was resting in bed during the afternoon.

Involving a significant other (SO) in assessing

An SO can be involved in the following ways:

- For elderly patient or those who cannot read/write, if the SO has been engaged in the counseling, we may discuss the option of the SO writing the activity calendar for the patient
- The SO can remind the patient at regular intervals (maybe once a day) to fill in the activity calendar and encourage them to do the planned activity that helps them feel better

Planning homework

Based on the activity calendar completed in the session, one activity that helps the patient feel better is identified. Discuss with the patient a specific plan for doing this activity at home. Encourage the patient to follow this activity at scheduled times till the next session.

In addition to doing the planned activity, encourage the patient to fill the activity plan (pg 23 of the patient booklet). Explain to the patient that noting the activity and rating the mood will help them see the relationship between doing activities and feeling better.

It is important to encourage the patient to anticipate possible barriers and to help the patient brainstorm ways to overcome these. The patient may report various barriers to doing the homework.

Table 8: Solutions to overcome barriers in doing homework

Barriers/challenges	Solution
Patient says he/she may find it difficult to remember to do the activity that helps them feel better or to fill the chart	Link the activity and completing the chart to some other routine activity such as meal time.<br>Use reminders such as stickers in a prominent place – on a mirror or in the kitchen, a phone alarm, etc.<br>Involve a SO to remind the patient
Patient says he/she does not have the time to do the activity and/or fill the chart	Help the patient identify a possible time when he/she may be more likely to do the activity and/or fill the chart<br>Ask the patient to fill it only once in the day rather than more frequently.
Patient cannot read/write	Help the patient identify a SO to fill the chart<br>Use the pictorial chart
Patient does not understand the reason to do the activity and/or fill the chart	Revisit the Healthy Activity Program, the link between activities and mood and the concept of activities that make them feel better and activities that do not make them feel better

Remind: *"I hope that you will follow the activity we have planned and also fill the chart and bring it when you come for the next session"*.

Summarising
As we did at the end of the first session, we help the patient summarise what we did together in this session.

Setting next session date
We arrange the time and place for the next session collaboratively with the patient depending on whether the patient prefers to see us at home, in the clinic or speak to us over the telephone. Make an entry in the phone diary.

Completing documentation
As we did in at the end of session 1, after the patient leaves, we need to complete the clinical form

Part 2: Getting active and solving problems

Goals:

- Strengthening the understanding of Healthy Activity Program and applying it to daily life
- Encouraging activation
- Identifying barriers to activation and learning how to overcome these
- Helping patients solve (or cope with) life problems

Outline:

- Reviewing progress
- Setting an agenda
- Reviewing homework
- Getting active
- Solving problems
- Involving a SO
- Overcoming barriers
- Planning homework
- Summarising
- Setting next session date
- Completing documentation

Introduction

This is the phase during which patients will learn most of the skills that they need to get active and address problems. The focus of this phase is on providing patients with opportunities for practice through systematic planning of homework.

Steps

Reviewing progress

This is done using the PHQ 9 and feedback is provided to the patient.

If the suicide item is positive on the PHQ 9 (i.e. item 9) then we need to assess and manage suicide risk as an immediate priority. If item 9 is negative, we may proceed to the next steps in the session.

Setting an agenda

Ask the patient what he/she would like to discuss during this session based his/her goals or recent problems or stressors.

Make a list of items to cover in the session such as:

- Asking about the homework the patient was to do at home – i.e. filling in the activity plan, carrying out the activities that were identified as targets.
- Reviewing the chart together and selecting activities that can help the patient
- Making a plan to perform these activities until the next session
- Discussing ways to deal with problems that the patient is facing
- Setting the next session date

The final step of agenda setting is to ask the patient if he/she is in agreement with the list and if there are any additional items that he/she would like to add.

Reviewing homework

In the third session, we can begin by asking,

"How did the homework go this week?" "Did you do the activity we had planned? Can you tell me you felt while doing it and afterward?

You can then ask regarding the activity plan

"Did you fill out your activity sheet this past week? Can we review it together now?"

To begin reviewing the chart with the patient we may ask them the following question:

"Before we talk about the activities that you have done, I was curious to know about your experience of filling the chart. What did you learn that you didn't know earlier?"

As we review the chart with the patient, some questions that may help us include:

1. What are the connections between the patient's activities and mood?

Does a patient show improvement or worsening of mood following the performance of an activity, for example, does the patient become very upset after talking with his/her relatives?

2. What patterns do we notice in the patient's mood?

Does the patient have a particular trend, for example, in which the patient reports better mood on Sundays than other days of the week? Or, does the patient feel better in the evening when the family is home rather than the rest of the day when he/she is alone?

3. How well do the patient's routine activities support his/her mood? And, are activities that used to be part of the patient's routine no longer present? For example, has the patient's routine activities i.e. sleeping, eating, bathing, exercising become disturbed leading to worsening of mood? Is the patient skipping meals, or not eating enough, this making him/her feel more tired and stressed? Did the patient use to do things regularly, such as physical exercise, that he/she has stopped doing?

In the fourth and fifth sessions the specific emphasis is on carrying out actions that were assigned. So, we can ask,

"What was your experience with arranging a meeting with your friend this week?" or, *"did you go for a short walk every evening as we had planned? Can you tell me more about it?"*

It is important for us to encourage any progress that the patient has made on the action plan and to review what was done (or not done) in detail.

Some barriers that we may face are:

Table 9: Solutions to overcome barriers in completing the activity plan

**Barrier**	**Solution**
Patient does not fill activity plan or says that he/she cannot fill the plan	Explore the reasons for not filling the plan<br>Explain the reason for filling the plan in detail again<br>Attempt filling the plan in the session
Patient writes an activity on the plan that is not specific like "at work" for large duration	Ask specific questions to define the behavior clearly - what does the patient mean by 'at work'. what is he/she doing, with whom, for how long, etc.<br>Share with the patient that you want to learn enough about their activities that you or someone else could repeat the patient's actions based on the description. For example, if a mother says that she was making dinner for her children, ask yourself, could you repeat what she did based on what you know? If all you know is "making dinner," probably not! However, if you learn that she made rice and fish curry, and also some chicken for a treat, then you have much more information. Many times patients report activities like "stayed in

	bed," but it is important to ask more so that we can describe what were they doing specifically.
Patient reports an activity you think might be causing problems (e.g., sleeping a lot), but you are not certain how important it is for the patient's mood.	It can be helpful to ask more about the frequency and duration of the activity that may be causing problems and whether there has been an increase or decrease in the activity. For example, a patient reports that he has been sleeping a lot, we may ask him: "*As you mentioned that you have been sleeping a lot, could you please describe to me how much is that? How much more do you sleep than your usual time? Also, how often do you sleep like that*? *How did you feel after waking up yesterday*?"
Patient reports feeling worse during an activity but you do not understand what led to the worsened mood	Ask more questions to understand the context of the activity. For example, a woman whose mother in law keeps on complaining about her cooking may report avoidance of the activity or increased anxiety whenever she enters the kitchen. So we may say: "*From what you tell me, you went into the kitchen deciding to cook a meal for the family but then lost interest and got into bed instead. Could you tell me what happened when you went into the kitchen?*" We may learn that the mother in law made some critical remark to which the patient reacted by giving up and going to bed.
Patient is did not carry out the activities as planned in the previous session despite attempting to do so	Review the activity plan and cut back on the number and/or frequency of activities. It may be that we are going too fast and the activation plan needs to be more modest. We may change the activity to something that is easier for the patient to do.

<u>Getting active</u>

Based on the assessment, we can select a few activities with the patient to structure and schedule for the action plan that will become the homework prior to the next session.

Selecting activities for the action plan:

The target activity is identified through a collaborative process of assessment that begins with the HAP model. Some important guidelines in choosing activities are listed below:

- Early in counselling, it is often helpful to start with activities that will help the patient feel better and that the patient is most likely to do, even if it is a small activity. These activities will have a greater chance of success and will encourage the patient to try other activities.
- These first actions may not address some of the primary problems in the patient's life, but will help the patient begin to feel more positive and thus be more prepared to tackle bigger problems in time. For example, a patient who does not have a job may come to us with complaints of sadness. Our long term target may be to help him/her get a job, but it is helpful to start working on what is immediately helpful to the patient, e.g. experience benefits of speaking to a friend or taking a morning walk.

Breaking down the activity.
In Healthy Activity Program, we focus often on helping patients to break down more complex or challenging behaviors into steps. Patients may find activities overwhelming and not know how to begin. Or, they may wish to achieve a lot in a short period of time.

We need to remind patients as well as ourselves to start small and build on the achievements that are made at each step. We can explain to them that by doing so they can slowly build their confidence, which will go a long way in attempting to achieve bigger goals.

Some examples of breaking down activities into small steps that the patient can engage in a week at a time include:

<u>Spending more time with family</u>
Return home from work an hour earlier
Sit and read a newspaper in the same room where his children and wife are playing.
Watching them while they play
Participating in play with them

<u>Establishing contact with old friends</u>
Make a list of friends who the patient would like to contact
Get their contact details e.g. telephone numbers
Identify who may be the easiest to contact
Choose a time/day of contacting them
Call the friend and have a conversation on the telephone.
Arrange to meet for a short while

Timing the activity.
We need to help the patient allot a specific time to do the activity. It may not be possible to schedule all the activities, but when possible, we help the patient identify the duration (when and how long) as well as the intensity (how much) of the activity that he/she is scheduled to perform. For example, if a patient wants to focus on doing housework such as cooking, it may be helpful to plan when he/she is going to do the activity (in the morning/evening), for how long (e.g. for 20 minutes or 40 minutes) and how much (prepare one or two items of food).
Troubleshooting difficulties in doing an activity
Finally, it is extremely important to identify possible difficulties to completing activities. Help the patient understand that he/she may not accomplish all of the activity despite best efforts because barriers may get in the way. Thinking ahead of the problems and planning for how to overcome them is an important part of developing effective action plans. The table below provides some guidelines for dealing with such barriers.

Table 10: Solutions to overcome difficulties in doing an activity

**Barriers**	**Solutions**
The patient says they may not have time to do the assigned activity.	Help the patient plan specific time and day they will perform the activity.

	Help the patient think of how they may reduce some of their other tasks and make time for the assigned activity
Patient says they may forget to fill the chart/ do the activity	Keep the plan in a prominent position such as kitchen or dining table.<br>Involve the SO in reminding the patient to complete the activity.<br>Set an alarm/reminder on a clock/telephone to remind the patient
Patient lacks confidence about doing the activity, says that they may feel too low/stressed.	Emphasize how the activity will itself help the patient feel better, go over the Healthy Activity Program briefly.<br>Explore with the patient who may be able to help him/her with the activity<br>Involve the SO in encouraging the patient to do the activity<br>Review the activity and break it down into smaller steps and encourage the patient to start with one step at a time.

Script 1

Who said?	***What did they say?***	***Comments***
Counsellor	Anjana, the last time you had shown me the activity plan and told some interesting things that you realized while filling it up. I wonder if it would be helpful for us to talk about that again. I think you had some really good ideas.	The counsellor starts the discussion in a manner that is very collaborative and encouraging of the patient’s progress in understanding the links between how she feels and what she does.
Patient	Sure, I told you that after I started having the problem, I stayed away from meeting my neighbours, basically cut down my social interactions completely.	
Counsellor	You are recognizing some important ways in which your activities affect how you feel. That’s great!	Again, the counsellor refers to the Healthy Activity Program, highlighting the connections between mood and behaviour that the patient is identifying.
Patient	I think I should start seeing my neighbours again. I can start spending some more time with them. What do you think?	
Counsellor	I like the fact that you are so motivated to improve, but, I’m wondering if you are suddenly taking on a lot of things.	The counsellor will have to actively work towards setting goals that are realistic and doable.

Who said?	***What did they say?***	***Comments***
Patient	Well, really? But, didn't you say that in this treatment, we need to force ourselves to do a lot of things to improve our mood.	Anjana and a lot of patients may not understand the process of activation.
Counsellor	You are right about activities having an impact on our mood, but it is not about forcing yourself to do things. I think the goal of getting connected with friends is very important but I wonder about breaking that down into smaller steps. I want to help guide you to some steps that you can take now and then we can build on those over time. Maybe start with something that has worked for you in the past?	The counsellor again encourages her but also helps her understand the idea of structuring activity (or breaking down larger actions into smaller, doable steps).
Patient	Well, I can drop in for tea with my neighbours.	
Counsellor	Did you go with them earlier?	
Patient	Yes, before I started having problems.	This is a promising place to start because it used to be part of her routine.
Counsellor	How often do you go? And, when?	Being specific about the frequency and duration of the action is important for scheduling.
Patient	We meet around 4 pm.	
Counsellor	Given your household responsibilities, is that a convenient time for you?	Here, the counsellor checks for difficulties, which will help her schedule a more effective action, plan.
Patient	Yes I am mostly free at that time.	
Counsellor	That nice. How long do you go for?	Again, the counsellor is being very specific in developing this plan. As she does, she identifies another way to break down the activity by structuring – guiding the patient to not start with too long of a visit. She wants the patient to have an experience of success as she begins!
Patient	Around 1 hour. But, I don't think I can do that.	
Counsellor	How about 20 minutes to start with and you can increase as you go along?	Addressing difficulties

Who said?	*What did they say?*	*Comments*
Patient	I can do that.	
Counsellor	Great! Are there any other things that might get in the way of doing this action plan?	Checking for difficulties
Patient	Well, maybe my children need me at that time.	
Counsellor	Can you take care of their needs beforehand and tell them that you will be going out for sometime so they are aware?	Here, the counsellor uses the problem solving strategy of brainstorming to suggest a solution to the problem of waking up in time interfering with the visit.
Patient	I think so.	
Counsellor	Great so can we put visiting neighbours for 20 minutes in the evening as an activity. Is there any other problem that you may have doing this activity?	Being specific about an activity is helpful to make sure that it is done.
Patient	No, I think I can do this.	

Solving problems

What is the importance of solving problems in patients with Depression?

Patients with depression often report difficult life problems that triggered or are maintaining their depression (see *Chapter 1*). The problems may be in the form of financial difficulties, relationship issues, health concerns or even difficulty in doing routine everyday activities like reaching the office on time. Patients also may talk about multiple problems that they are facing in their lives. The patient's experience of these problems is often closely linked to their symptoms. Thus, problem solving is an important form of activation for many patients.

The steps to solve problems (*Figure 1*) can be used across a range of activities to address multiple problems. Some of the issues that patients face cannot be changed easily (like an abusive spouse, natural calamity); however their reactions to these events and the way they cope with them can often be addressed using the problem solving in a stepwise manner.

How do we help solving problems with patients with Depression?

Overall, solving problems involves helping patients:

- Identify problems that are contributing to their depression,
- Generate multiple solutions,
- Apply the most appropriate one and
- Review the effectiveness of the chosen solution.

We work with patients to identify and then solve problems in a systematic 6-step process (*Figure 1*). Although we may not follow each step strictly in each session, these general steps guide the counsellor in helping the patient to take an active, problem solving approach to challenges in his/her life. It is useful to start problem solving once we have a fair understanding of the problems and the activation has begun. However, if the patient is overwhelmed with their problems, it may be necessary to address the problems directly before the patient focuses on increasing pleasurable activities. We can say,

"It seems like some of the problems you are dealing with are making you feel overwhelmed and preventing you from doing the activities that you enjoy. Is that correct?"
We can then add, *"Let us discuss some of the problems and see how we can work together to help you solve these".*

Figure 2: Steps in solving problems

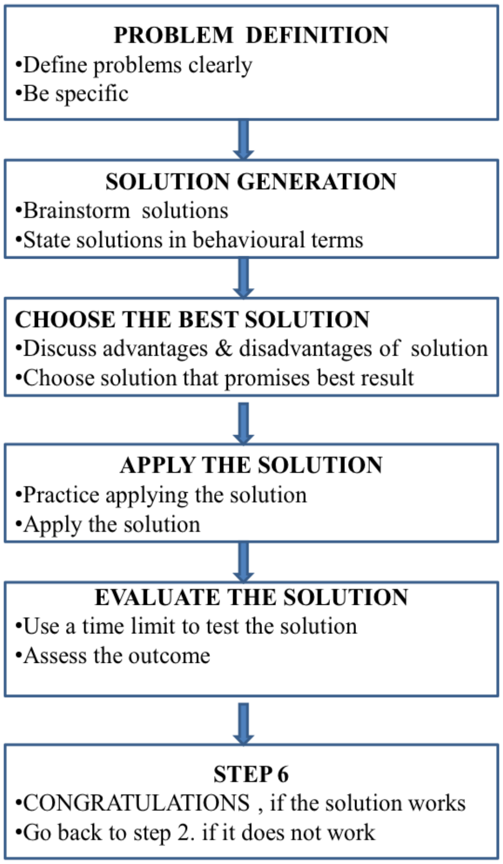

Script 2

The case of Anjana may help us understand how to use problem solving effectively.

Who said?	***What did they say?***	***Comments***
Counsellor	I'm glad you could come for the session. I was hoping to meet you after the screening in the clinic.	
Anjana	I had to come. My life is so bad that I can't take it anymore.	
Counsellor	Would you like to tell me more about it?	Open ended question

Who said?	***What did they say?***	***Comments***
Anjana	Nothing works, nothing at all works. I deal with one problem and another one comes up, you deal with that and then…. It never really stops. My husband has a drinking problem, there are financial pressures and I cannot even keep up with my job. I don't know what you can do, I'm not even sure if this will work.	
Counsellor	I can see that a lot of things aren't going well for you. But, I get a sense from our sessions so far that the financial pressures seem like the most terrible thing (*Patient nods to say yes*). Tell me more about it.	**Problem definition**<br><br>Counsellor begins to be specific
Anjana	I have been dealing with financial problems for a long time but somehow I have managed. Now my children are getting older and they have more needs and I am feeling the burden.	You may have to help them become specific.
Counsellor	And your husband….	
Anjana	Oh yes! he can see that things are more difficult and I am not keeping well, but he is adding to my burden by continuing to burn money on his drinking.	
Counsellor	So, it seems like working together on the problem of your financial issues is very important for helping you feel better. Is that right?	An example of problem definition
Anjana	Yes, definitely. It's my biggest stress.	
Counsellor	Now that you are more active in your daily routine, of visiting your neighbours, do you think this is a helpful time to start working on the financial problem?	Questions to guide solution generation in a collaborative manner
Anjana	I do, I do. But I don't know what to do about it. I can pick up another part time job but I don't feel too well. Maybe I can talk to my brother and ask him to help me out, but I don't know if he will.	

Who said?	*What did they say?*	*Comments*
Counsellor	Let's see if we can work together on this. Let's slow down as we start and just concentrate on looking at the options. Let's get a bunch of options on our list and then we can see which one or ones might be most promising for solving this financial problem. You have already listed a couple of options –Taking up another part time job and asking your brother for help. Is there anything else you can think about?<br>How about looking at your monthly expenses and seeing if you can save somewhere. Can you think of something on similar lines?	In solution generation we do not evaluate our solutions but keep the focus on getting as many solutions as possible.<br>Also, it is ok for the counsellor to make suggestions for possible solutions and to discuss these with the patient. Notice how the counsellor asks Anjana about potential solutions as opposed to telling her what to do.
Anjana	Alright, in that case, I can see for a month. That's all I can think of...	
Counsellor	Well, I think that is quite a lot (Smiles). If you look at all the solutions, which would you prefer the most. In the entire list that you have successfully made, which one do you think you can do most comfortably?	**Choosing the best solution**<br>It is important to validate a patient for the efforts made by them. After the patient has generated the solutions, she can be guided to choose the best one. The choice of solution is guided by one that is most likely to be successful, improve the patient's symptoms and is in line with the patient's goals.
Anjana	I think, looking at one month's expenditure and re-working the budget.	
Counsellor	How helpful would that be?	It is important to help patients see the pros and cons of their chosen solution.
Anjana	It will work well. Sometime I do think there is wastage of money and if there is better planning, I can save and spend on other important needs.	
Counsellor	What made it hard to assess this at that time?	Explore barriers in applying solutions
Anjana	I never keep a written account so I forget and then it becomes impossible to recall at the end of the month.	
Counsellor	I may have some suggestions for that like maybe you can keep a diary and at the end of every day spend 5 minutes to write down the daily expenses…so you don't forget? Will that help?	**Apply the solution**

Who said?	*What did they say?*	*Comments*
Anjana	Yes, I could place the diary in the kitchen so that I don't forget.	
Counsellor	That's good. What else do you think you need to do?	Note the focus is on what to do, solutions have to be defined in behavioral terms
Anjana	I think, I will start with this and then see how it goes.	
Counsellor	(Smiles)I know, sometimes being specific helps. Can we set a timeline for this? When so you think you would like to assess the expenses?	
Anjana	I think I will try to do this for 2 weeks and see	
Counsellor	Ok, great. Let us then see how this works out. It always helps to test whether our solution is really helpful or not. I'm not saying that it will not work, but we need to be open about the result.	**Evaluation of the solution** Testing a proposed solution within a specified time frame is important to see how effective the solution actually is.
Anjana	Yes, ok.	
Counsellor	After which, just in case, re-working the budget does not work, you will try your next best option that would be...	The last step involves preparing both for the success and failure in the application of solution.
Anjana	I think contacting my brother is a good idea. He is usually very supportive. He also gives me good advice when I need it.	

In addition to helping patients solve the current problem/s in their lives, it may be helpful to teach patients these steps so that they can use them for problems that may arise in the future. We may say,

"Can we go over the steps we have followed to help you with your problem of feeling sad and low (or stressed about money, etc.)? We first defined the actual problem you can work on, i.e. financial pressures, we then discussed a list of solutions, thought about the advantages and disadvantages of these solutions, identified the most promising one i.e. looking at the monthly budget and assessing if you can save on unnecessary expenses, and developed an action plan of keeping a daily diary of daily expenses which you will try out in the coming days".

In this way, we are teaching the patient a new skill that she/he can use when faced with problems in the future.

Sometimes, the patient's problems may be difficult and/or multiple, and we may find it challenging to generate a list of solutions. The table below provides a list of some common problems that our patients struggle with along with some possible solutions.

Table 11: Possible solutions for common problems

Problem	Possible Solutions
Family member has a drinking problem	• The concerned person can be encouraged to seek treatment. We can give the patient a brochure about hazardous drinking that they can share with their family member<br>• Persons close to the family member and whom he trusts/respects can encourage and support him to stop drinking
Husband is physically abusive towards the patient	• Seeking the help/support of someone who may be able to speak to the husband<br>• Discuss safety measures<br>• Referral to agencies that can support the wife
Patient does not have a job	• Explore options of job opportunities like ads for vacancies<br>• Asking friends and family to explore their network<br>• Sharing information about government employment schemes
Illness in the family	• Encouraging the family to seek the right treatment<br>• Follow medical advice<br>• Referral to a specialist/agency/hospital<br>• Caring for the person's needs adequately<br>• Seeking support oneself to deal with the burden of care
Relationship difficulties	• Identifying ways of communicating better<br>• Asking the person close to patient to come for a session<br>• Involving a third person who can help the patient
Financial difficulties	• Looking for better job opportunities<br>• Referral to government schemes such as pension schemes<br>• Explore ways of saving<br>• Seeking help from friends/family
A person in the family with special needs	• Seeking professional help to support the person with special needs<br>• Gather information and enhance skills to care for the person with special needs<br>• Seek support oneself to deal with the burden of care<br>• Referral to agency
Difficulty in coping with work stress	• Learning effective ways to cope with stress such as taking short breaks<br>• Time management<br>• Enhance skills that will help with work

	performance<br>• Seek support from seniors/co-workers
Stress and loneliness caused by living away from home and family	• Maintaining regular communication with the family<br>• Creating a support network of friends<br>• Join community activities
Elderly patients with no family support	• Explore possibility of building a support network<br>• Provide information about government schemes<br>• Referral to agency

Involving a significant other (SO) in activating and solving problems

The SO can be involved in the following ways:

- Help identify activities that make the patient feel better and those that do not make the patient feel better in addition to what the patient may have reported
- Help identify a problem and generate solutions when attempting to solve problems in the patient's life
- Support the patient in doing the activities, for example, if a patient cannot get out of bed in the morning, her husband can help by reminding her to set the alarm the previous night and waking her up when it rings in the morning
- Help the patient record the activities in the chart when the patient is not keen or unable to do this
- Support the patient in coming for sessions regularly and accompanying the patient if required

Planning homework

The homework will focus on helping the patient get active with doing activities that make them feel good and/or doing activities related to solving problems. We develop the specific homework collaboratively based on an assessment of the links between activities and mood and of the problems the patient is facing currently.

We list out the agreed activities in the activity plan (pg 23 of the patient booklet) and the patient puts a tick when they perform the activity during the week. They also record their mood when doing the activity (as described under session 2). If the patient has not completed the activity plan at home, it is important to fill it in the next session.

Remember: The emphasis is on doing the activity, which is given as the homework. It is helpful if the patient is able to record it, however, if the patient has not completed the chart, it can be done in the session.

We also measure the degree of activation by listing out the activity/activities and asking the patient to share feedback on the extent to which the activity/activities were preformed on a 4 point likert scale in the Clinical Record Form.

It is important to plan for problems or barriers that may get in the way of the patient doing the activity. It is helpful to help the patient understand the fact that he/she may not accomplish all of the activity despite best efforts because barriers may get in the way.

Summarising

As we did in the previous sessions, we help the patient summarise what we did together in this session.

Setting next session date

We arrange the time and place for the next session collaboratively with the patient and enter this in the phone diary.

If the patient is not keen on a home visit, arrange for a suitable day and time for them to come to the clinic or confirm that we will conduct the session on the phone.

If this is the last session of Phase 2, it is important for us to remind the patient that the next session will be the last session of the treatment. This may make some patients worried about how they will cope without counselling – we need to reassure them by pointing out various ways they have learnt to deal with their problems through the process of counselling.

Complete documentation

After the patient leaves, fill the clinical record form.

PHASE 3: ENDING WELL

Phase 3 is delivered in one session

Goals of Phase 3:

- Help the patient review the HAP model in general and specific actions that support the patient's mood
- Help patient identify possible challenging future situations
- Help patients make a plan to deal with such situations using the skills they have learnt

Outline of Phase 3:

- Reviewing progress
- Setting an agenda
- Reviewing homework
- Reviewing skills that the patient has learnt
- Preparing to stay well over time
- Summarising
- Completing documentation

Introduction

The last phase can occur at any point after session 4. We can end treatment if the following criteria are fulfilled:

- The PHQ 9 scores in the last 2 sessions have been 9 or less
- The goals of the treatment have been met (for example, the patient is active, the patient has begun to take steps to solve problems, etc) as agreed upon by the patient, the counsellor and the supervisor.

Ending well aims at identifying potential triggers that may increase the risk of having another depressive episode in the future and effectively addressing these by reviewing what has helped during counselling and how to continue to take such actions in the future.

Steps

Reviewing progress

This is done using the PHQ 9 and feedback is provided to the patient.

If the suicide item is positive on the PHQ 9 (i.e. item 9) then we need to assess and manage suicide risk as an immediate priority. In such cases, the counselling sessions will need to continue further in discussion with the supervisor. If item 9 is negative, we may proceed to the next steps in the session.

Setting an agenda

- Ask the patient what he/she would like to discuss during the session
- Make a list of items that we would like to cover in the session such as :
    - Asking about the homework– i.e. activation, solving problems
    - Reviewing what skills the patient has learnt and activities that can make him/her feel better
    - Summarizing steps to help support the patient in staying well over time

The final step of agenda setting is to ask the patient if he/she is in agreement with the list and if there are any additional items that he/she would like to add.

Reviewing homework

We can ask

"How did the homework go this week?" or *"What did you learn from doing the activities we had planned during the last session?"* or *"What barriers did you face in doing the activities we planned in the last session?"* Or, we can ask about specific activities, *"What did you notice in doing the steps we had discussed to reduce the quarrels with your wife?"*

Reviewing the skills that the patient has learnt

- We may ask:

"As this is our last session, I would like to know from you what it is that you have learnt from these sessions that we have had together? Can you tell me in your own words what are the most important things you have learned?"

- Clarify information that is not clear. It is useful to revise the HAP model. We may say:
  "We also discussed how your activities can affect how you feel and how they can make you feel better or can make you feel stuck or stressed."
- It is very useful for us to highlight specific actions that the patient used to overcome depression that may not be clear in the patient's memory. For example, we may say,

"When you felt down, we noticed that taking a walk in the morning was very helpful for your mood. Do you agree?"

- Emphasise the patient's role in getting better. The patient may fail to recognize the efforts he/she has made and attribute their improvement to us. It is therefore important to remind them of this.

"You have made a lot of effort towards recovering from your stress. Despite feeling like you didn't want to get out of bed in the morning, you gradually managed to not only wake up early every day but also do all you needed to help prepare your children for school. One of the ways you did this was by starting with a single step (waking up every day) and then building on that with harder steps over time (like making breakfast). You also asked your sister to help you with some of the household tasks until you felt well enough to do these yourself".

- Motivate patients to use the strategies across other life situations

A patient seeing us may often be of the opinion that the skills learnt to solve a particular problem may apply to that problem only. In such as case, it can be helpful to encourage the patient to identify and apply the skills across different situations.

For example, a patient who has learnt actions to help solve quarrels with a spouse may be encouraged to use it in other contexts like interaction with seniors at the workplace and other family members.

"Do you recall how you approached your friend for help in dealing with the problems you were facing with your wife? Talking with him was very helpful to you in feeling encouraged and practicing how to make your wishes known to your wife. Do you think that taking this same action could be helpful in dealing with your current problem of how to tell your boss that you need some time off work to attend to family matters?"

Summarizing steps to help the patient stay well over time

This is done by filling in the End of Treatment form with the patient in the session. This involves the following steps:

- List the activities that the patient needs to maintain or increase

It is important for the patient to continue to stay active in ways that have helped. For example, speaking to a friend regularly, cooking meals that the children enjoy, and visiting a relative. Go over the activities that the patient has found useful during counselling and list these down.

"Over the time we have been meeting, there are activities that you have begun doing that have helped you feel better. Can you tell me what these are? Let us list here together at least three of the most helpful one's for you."

- It also is important to identify situations that might make it harder to keep doing activities that make them feel better. Ask the patient what situations might increase her/his risk of developing tension or Depression in the future.

"What situations might arise in the future that make it hard for you to keep doing activities that make you feel better or that might increase your risk of tension or stress? Let us together list here at least three of the most difficult ones for you."

Explain to the patient that knowing the stressful situations in advance can help them to take action to protect themselves from tension as much as possible. In addition, though, patients need to identify what they may do or not do in such situations that make things harder.

- What are the down activities that the patient may start doing again?

The patient is alerted to the early warning signs of depression. For example, staying alone and reducing social interaction, neglecting their diet and not eating regular meals, neglecting routine household work. Prepare a list of the activities that do not help the patient feel better with the patient.

"What are the things that you do or don't do when you are starting to feel down that make you feel worse? Let us list here together at least three of the most challenging ones. They might be things like staying in bed more, not talking with your sister, and so forth. "

- What can help when difficult situations arise?

For example, breaking down the activities into small steps, seeking the help of an SO in doing the activities, maintaining an activity plan

"So if you begin to feel the same way (or do the same things), how will you be able to do the activities that will make you feel better? What are the ways in which it will be easier to do these activities (refer to activities listed above)?"

It is important for us to be as specific as possible when making this list with the patient. We can think of it like writing down a recipe for good health. If we were to follow the steps, would the list of ingredients and instructions be clear enough to produce the meal we want to? If not, we talk more with the patient to make the action steps as clear and specific as possible.

<u>Involving a significant other in staying well:</u>

An SO can be involved in the following ways:

- Encourage the patient to continue engaging in activities that help them feel better
- Identify any stressors that may cause tension and help the patient find solutions to deal with it

<u>Summarising</u>

As we did in the previous sessions, we help the patient summarise what we did together in this session.

After the patient says what he/she has learned, repeat his/her ideas and add these things:

"In this session:

- *We have looked back on what you have learnt during counselling to help you feel better. We have identified possible situations in your life that may make you feel stressed again*
- *We have made a plan of how you will use the skills you have learnt to deal with these situations"*

It is important for us to summarise the patient's efforts during the counselling, thank them for their participation and ask about any remaining questions or concerns and remind them of contact details in case they need additional help.

Remember:

If a patient remains unwell at the end of eight sessions, we refer him/her to an independent psychiatrist who has been contracted for this purpose by the program.

If a patient who has dropped out returns, we offer counseling and continue from the last session.

While a patient who has been discharged returns seeking help s/he is encouraged to consult the PHC doctor for his/her difficulties or referred to the psychiatrist. NO further session is offered.

Complete Documentation

After the patient leaves, fill the clinical record form and the end of treatment evaluation form.

Chapter 5

Useful Strategies For Specific Problems

Learning Objectives

In this chapter, we will learn:

Ways to deal with 5 common problems that can make it more difficult to get active and solve problems:

- Thinking too much
- Feeling anxious or tense
- Problems with people close to the patient
- Difficulties with sleep
- Using tobacco

STRATEGIES FOR THE PROBLEM OF "THINKING TOO MUCH"

Does the patient tend to think over and over about their problems and the cause of such problems? Does the patient tend to get caught in thinking negative thoughts, which worsen his/her mood and lead to more negative thoughts?

People suffering from depression often focus on thinking about themselves and how they feel, the condition in which they find themselves, and the reasons for their struggles. Often patients describe this experience – repeating thoughts about these topics over and over again in one's mind – as *thinking too much*.

The problem of thinking too much can make it hard for some patients to do activities. At times, their main activity might be just that – thinking too much. Others do activities but they report that they do not feel any better. When we ask more about their activities, we learn that they were thinking too much while engaged in activity.

For example, if a patient reports going for a scheduled activity like a walk in the park, but reports feeling low the entire time, it can be helpful to ask what was going on in the patient's mind. If the patient reports that she was repetitively thinking about her fight with her husband, such thinking may be an important target of intervention for two reasons. First, the thoughts may be preventing the patient from taking pleasure in activities like walking in the park. Second, the thoughts may be preventing the patient from effective problem solving or taking steps toward change; she may believe that thinking more about the conflict with her husband will lead to new solutions, even though it keeps her stuck in feeling down.

How can we help patients who are caught in thinking too much?

We can use activities to decrease thinking too much. Using activities to address thinking too much is a process that requires time and effort both from the patient and us.

First, it is important to identify and highlight the effects of thinking too much:

- Thinking too much may be such an automatic behavior that patients may engage in it without awareness of its impact. We can help patients see the effects of thinking too much on how they feel. We may say,

"Anjana, when you are in bed thinking again and again about the pains in your back and the demands at your job, how do you feel? What about when you get up to prepare dinner for the family? Do you feel more or less tired?"

In these ways, we can begin to help patients understand the impact of thinking too much about how they feel.

- Patients also may believe that thinking too much is helpful in overcoming their problems. We can help patients see the effects of thinking too much about the problems in their lives. We may say,

"*Anjana, we have talked about how you stay on your own and think again and again about your husband. It does not appear that this is helping you to solve the problems you and he face. Would you agree with that? I know it is hard to change the habit of thinking too much, but one of the first steps is noticing that it does not often solve your problems – even though it seems like it will.*"

- It may be important to help patients learn to "catch" themselves when thinking too much and notice its effects in that moment. We may say,

"*Anjana, the first step in changing the problem of thinking too much is realizing when it is happening. This week, I would like to suggest that you notice one time each afternoon when you are thinking too much and how you feel in that moment. Would you be willing to practice with this?"*

Second, it is important to guide the patient in some alternative actions to take when they find they are thinking too much. There are 3 main options in dealing with thinking too much:

Help the patient solve the problem

It is often valuable to help define the problem that the patient is thinking too much about and then outline steps towards active problem solving (see *Chapter 4*). For example, we may teach Anjana to define the problems that she thinks about over and over and work on solving these problems in our sessions, focusing on how to talk with her husband in more useful ways, how to get support from her neighbor, and how to reduce demands at work.

Teach the patient to attend closely to sensory experience

- Thinking too much automatically shifts the patient's focus from the present moment to the mental tape that is going on in their mind.
- We can teach the patient to refocus attention to the present moment by attending to a specific sensory experience in the present moment such as sounds, smells, visuals, tastes, or touch.
- To teach this strategy to the patient, do the following:
  - First explain the purpose of this strategy:
    "*We will be learning some homework today that can be helpful when thinking too much is a problem. It is a way for you to practice keeping your mind focused in the present moment by directing your attention to what you feel, see, smell, hear, etc.*"
  - Second, ask the patient to focus on one sensation as you guide them in practicing the strategy in the session. For example, we may ask the patient to focus on the room in which we are sitting, and to describe all of the colors that they see in the room.
  - Third, ask the patient about he/she experienced and answer any questions or clear any confusion.
  - Fourth, ask patients to practice at home by developing a plan for when and how long they will practice. The time and place can be linked to the settings in which the patient is most likely to be thinking too much, but it also can be helpful if the patient starts to practice at times that are not the most challenging in order to build their basic skill. Also, remember to ask about what might get in the way of practicing and solutions that might help.

Distracting

- Distracting oneself from thinking too much helps shift the focus to something new or different in the environment. This helps to distract their attention from thinking too much. Some helpful activities to explore with the patient include: physical activities (brisk walking, fast paced household chores), activities that shift location (visit a neighbor, walk outside), activities that are engaging (watching a funny movie, talking with a close person).
- It is possible to plan in advance for such activities by identifying the context in which patients commonly think too much.
- Most of our patients report using distraction activities at some point during their days. Our role in the session would then focus on guiding them to use this strategy as an alternative to thinking too much.

Refer the patient to the relevant section of the patient booklet that describes how to deal with thinking too much. The patient can read this at home (or ask a significant other to read it for them). This will remind them of the strategies they can use to feel better.

For example:

Script 3

Who said?	*What did they say?*	*Comments*
Counsellor:	Anjana, you seemed quite upset yesterday when you called. What had happened?	
Patient:	I have been doing all the activities that you had asked me to do. As a matter of fact, now I try to be as busy as I can. Even if I'm tired, I keep doing things. It's been 3 weeks but I haven't improved.	
Counsellor:	I can see you are quite upset about the lack of improvement. But, I was keen to know about what you said about keeping yourself very busy. How do you do that?	
Patient:	We had discussed a list of activities; you had given me a chart to write it in. I do that, but it still doesn't help.	
Counsellor:	Do you have the chart with you? (*Patient hands over the chart*) Alright, if I take this activity of visiting your neighbours, can you tell me more about it?	
Patient:	Well, I go over to meet some of the neighbours who get together in the evening at one of their homes	
Counsellor:	That is great that you are being active in these ways that we discussed. That is very hard to do and you are doing it! Let's look more closely at what you were doing at that time so we can understand better how you are not feeling improved. You said you visit one of the neighbours' home. Would you describe what is going on when you are sitting there?	It is important to provide encouragement as patients begin to get more active. It also is important to assess what is happening so you can intervene.
Patient:	Well, I look at all the ladies who get together and they are talking and laughing. I think of how happy they are and then I am reminded of my troubles and how difficult my life is. I wonder if I will ever be able to enjoy myself like them. Then when I get back home, I feel more tired than fresh. To stop thinking about all of this, I've been working harder but that doesn't change anything for me also. I just feel more tired.	The patient is describing the ways in which she was engaged in two activities at that time. One was being with neighbours and the other was thinking too much about her unhappiness. It is critical to identify that thinking too much is present so that you can work on targeting it for change.

Who said?	***What did they say?***	***Comments***
Counsellor:	I see how hard you have been working to keep up your mood. I also know that it feels really hard, when improvements don't come by. Though I can't help noticing that even as you work really hard, try new activities and keep doing things, your focus is often on the thoughts that you have. So, you may be visiting your neighbours but at the same time you are also very busy with thinking too much. In this instance, I am wondering if thinking about your unhappiness was keeping you caught in feeling down. Does this seem right for you?	The counsellor checks out her ideas about the effect of thinking too much on the patient's mood. It is important to assess whether our ideas about what is keeping a patient stuck fit with the patient's experience.
Patient:	Yes, I keep getting the thoughts. It's like a film that keeps running in my head. How do I deal with that? I sometimes think about something else, like happy memories related to my children. It helps for some time, but then I'm back to feeling upset.	The patient confirms the counselor's theory, which helps them target thinking too much
Counsellor:	It is often these thoughts that keep us from being engaged fully in the activity that we are doing. I have some ideas of things you could try to deal with this problem of thinking too much.	It is important to provide information and hope.
Counsellor:	We can think of how to increase your awareness of the present moment. What happens, when we think too much, to use your words, when a film or a tape plays on in our head, we sometimes move away from the present moment. When this happens, we may do something mechanically but are not totally present in the here and now. You can increase your awareness of the present moment by attending to, let's say, the decorations in the room or the sounds in the room. What do you think about that option?	Suggesting a technique to address thinking too much
Patient:	Well, I can only notice the laughter and happiness in my neighbours' voices and that makes me think more about things and it would be hard for me to just pay attention to the surroundings in the room.	
Counsellor:	That makes sense. In that case, we might want to start with a different option. This other option is to focus on a distraction that might engage your attention easily. I wonder if you can help the hostess with making some tea or serving snacks?	One has to be careful while suggesting an activity. Look for a good fit between any of the three techniques and the patient's requirements.

Who said?	*What did they say?*	*Comments*
Patient:	Well, sounds better than just thinking, but I'll have to try it out.	
Counsellor:	Perfect! That's all we want right now is to try it out and we can learn from what happens and then revise the plan if needed.	
Patient:	Sure.	

STRATEGIES FOR THE PROBLEM OF FEELING ANXIOUS AND TENSE

Many patients with Depression report feeling anxious, tense, or stressed or that they have difficulty relaxing. These experiences are common among people who are depressed. Sometimes, people experience a lot of tension as they begin to get active. Excessive stress or worry can be a barrier to getting active and reaching out to others in one's life.

Different people find different techniques useful for coping with anxious/tense feelings on a daily basis. Some people report benefits from regular meditation, some find benefits from practice of yoga asanas and prayers, and so forth. It is helpful to review in detail what coping methods have been useful in the past for them during stressful times. One strategy that can help patients overcome this barrier is to practice specific relaxation strategies.

In this chapter we will focus on one type of relaxation training that involves deep breathing.

Our role is to teach the practice of deep breathing during the session, correct any mistakes that the patient may be making while doing the breathing exercise, assign the practice for homework, and review the patient's home experience during the next session, again providing feedback as needed.

It is important to note, however, that some types of anxiety require additional intervention and are not likely to be helped only by teaching relaxation. If the patient's anxiety is severe and persistent or occurs in brief sudden episodes, then it is important to refer the patient to a specialist for further management.

Steps

<u>Assess the need for relaxation training</u>

To determine if relaxation training would be helpful, we can ask these questions:

- *How long have you been experiencing these symptoms* (like bodily tension, palpitations etc.*)? How much trouble do they give you?*
- *When and where do these symptoms become most severe* (in the morning/ evening; at the office)?
- *What did you do to deal with these problems earlier? How helpful were these methods?*
- *Are you interested in learning a new method that focuses on breathing?*

Answers to these questions will help us structure and schedule the breathing exercise in the patient's routine.

<u>Teach the breathing exercise</u>

We guide the patient through the steps during the session.

Then, we let the patient continue the breathing exercise in silence for about 3-5 minutes.

We then ask the patient about what he/she experienced in order to identify questions, problems, or confusion. If necessary, we guide the patient in the practice again.

Once the patient has learned the exercise in the session, we assign it for regular home exercise. While doing this, we can discuss with the patient, the duration, frequency, place and the possible difficulties in doing the exercise.

- *Posture for the exercise*:
  There is no special position; any position that the patient finds comfortable is the right one. The patient can therefore sit or lie down (if there is space in the room).
  Give the patient a choice of doing the exercise either with eyes open or shut.
  Generally, the exercise is practiced with the eyes closed but some patients may feel uncomfortable and can leave their eyes open.
- *Breathing*:
  After about 10 seconds, the patient should start concentrating her mind on the rhythm of his/her breathing. Tell him/her to concentrate on breathing slow, regular, steady breaths through nose.
  If a patient asks how "slow" the rhythm should be, you can suggest that he/she should breathe in until he/she can count slowly to 3, then breathe out to the count of 3 and the pause for the count of 3 till he/she breathes in again.
  As the exercise progresses, the rhythm can be slowed even further according to the comfort level of the patient.
  We can suggest that each time the patient breathes out, she could say in his/her mind, "relax" or an equivalent thought in the local language. Patients who are religious can use a word that has some importance to their faith e.g., a Hindu could say "Om" while a Christian may say "Praise the Lord".
  Continue the breathing for at least 10 minutes until the anxiety has reduced.
  If a patient complains of palpitation, tingling - numbness in fingers or mouth, chest pain or any other physical discomfort during the exercise, it may mean that he/she is breathing too fast; slow down the rhythm to a rhythm that he/she finds more comfortable.

Refer the patient to the information about relaxation provided in the patient booklet (pg 8). They can read this at home (or ask a relative to read it for them) to remind themselves of how to do the exercise.

Script 4

Who said?	***What did they say?***	***Comments***
Counsellor	Anjana, the last time that you were here we spoke about how you were having difficulty at work, and how you always worry that you will not complete your tasks.	
Patient	That's right. I worry even if I'm not working … about everything. I can't seem to relax at all. I also feel tightness, like not feeling comfortable in my chest.	
Counsellor	So, when you feel like that, what do you do?	Assessing what the patient does to deal with their anxiety.
Patient	I try to think of something else, or listen to music. It helps for some time, but then again I start feeling uncomfortable.	

Who said?	***What did they say?***	***Comments***
Counsellor	Is there a time when it's really bad?	Assessing the symptoms in detail
Patient	In the morning when I begin work, I become really nervous. I keep thinking that I'll never be able to finish everything.	
Counsellor	That must be making you feel very anxious.	Reflecting feeling
Patient	It does, if there was any way in which I could stop all of this I would.	
Counsellor	Have you ever tried any relaxation exercises like breathing?	
Patient	I've heard about them, but I have never really tried it.	
Counsellor	Well, I am curious to know if you would be interested now (Patient nods). What you would be learning is a simple breathing technique that is used not only in medical clinics but also in yoga and meditation. This technique if practiced regularly has been found to be very useful in helping a person to relax. But, before we begin I would like to know that on a scale of 1(no anxiety)-10 (very anxious), how would you rate your anxiety now.	Getting an objective measure of anxiety is helpful
Patient	I'll say around 7-8.	
Counsellor	Firstly, I would want you to sit as comfortably as you can and gently close your eyes. (*After 10 seconds*) Now, breathe slowly and steadily from your nose, as you do that, bring your attention to your breath. You should breathe so slowly that you can count till 3 as you breathe in and till 3 as you breathe out. As you breathe out, you can also say to yourself "relax" "I'm feeling calm" "the tension is slipping away". Continue doing this till you feel completely relaxed, let's say stop when you think your score is 1 or 2.	Giving instructions for relaxation<br><br>You can either ask the patient till their score reduces or for a maximum of 10 minutes.
Patient	This feels better.	

Who said?	***What did they say?***	***Comments***
Counsellor	I’m glad! This something that most people need to practice regularly and you are off to a great start. Are you willing to practice this at home too? If so, can we go over how and when you will do this at home?	
Patient	Sure.	

DEALING WITH PROBLEMS WITH SOMEONE CLOSE TO THE PATIENT

One source of tension for many patients with Depression is the challenge of communicating effectively with other people in their lives. Some depressed patients have difficulty asking other people for help and this makes them feel isolated or overwhelmed by their problems. Others have difficulty saying no to other people's demands or requests and thus find themselves doing things they do not want to do, feeling pressured, or overwhelmed.

If a patient lacks the skills in communicating effectively with others, one activity to practice at home is communicating with close others. You can teach the patient the steps in the session and then ask if she is willing to practice at home. There are four steps:

1. Problem Solve: The first step is helping the patient to identify clearly what he or she wants or does not want. Thus, the patient who is feeling hopeless about a conflict with her daughter may be encouraged to identify what she would like from her daughter (e.g., wanting her to visit more often).
2. Communicate: The second step is teaching the patient how to communicate this clearly and effectively.

This can be done by remembering the acronym "DEAR":

Describe the situation briefly – what is happening?

Example: "We have been arguing so much lately about how rarely you come to see me".

Express feelings about the situation **-** don't expect the other person to read your mind.

Example: "I feel very unhappy about our quarrels and am also worried that our relationship will just keep getting worse"

Assert **-** Ask for what you want (or don't want) clearly.

Example: "I want you to come and have lunch with me every Sunday"

Reinforce – encourage the other person to support you.

Example: "This will make a big difference to how I feel. It would give me a chance to speak to you about what is bothering me and make me feel less lonely"

In order for DEAR to be effective, the following tips may be useful to improve the patient's communication skills. Refer the patient to page 6 of the patient booklet while explaining these tips. Often, it is helpful to:

- Focus on the current quarrel and not talk about all the mistakes the other person has made in the past.
- Separate the other person from his/ her behaviour. Using the words "*Your words were very hurtful"* lead to more constructive discussion than using the words "*You are an unkind person*".
- Acknowledge the other party's expectations. He/ she could say: "*I know you feel like I am not paying attention to you*".
- Use "I" statements about how s/he feels and what s/he wants. For e.g. you could say "*I feel angry when you behave like this*" rather than "*You make me angry*".
- Avoid using words such as "always" and "never". For example "*you never listen to me*" or "*you always shout when things don't go your way*".

3. Practice: The third step is creating opportunities to practice these skills in action. We can do this in the session using role play. For example, saying, *"I will be your daughter, ok? I'm calling you on the phone now. Hello mother, I can't visit you this weekend. We're really busy. Maybe I will come next weekend, ok?"* The patient is then asked to respond in the way described above. If the patient doesn't respond, she may require more coaching. *"So what if you were to say…"* and then continue the role play with the patient so he/she has an opportunity to practice saying those words.
4. Schedule a session with the person concerned. Ask the patient to find a good time to talk when the other person is more receptive to the conversation. It is easier to practice if neither person is angry or upset or in a hurry. Also, if the patient prefers, a session can be scheduled where the person concerned is invited to join and the patient is encouraged to

communicate based on the above steps. The patient may feel more confident and supported while in the session and you can assist as the need arises. It is also possible to discuss finding another helper if s/he cannot directly communicate with the other person. This can be someone both of them have trust and respect for.

DEALING WITH DIFFICULTIES WITH SLEEP

Many patients with Depression will have poor sleep. This may take many forms:

- Inability to fall asleep leading to lying awake in bed for hours at night
- Waking up frequently during the night and being unable to sleep again
- Waking up much earlier than one's usual time and being unable to fall asleep again

Inadequate sleep leads to the patients feeling even more tired, can interfere with activities and further worsens the mood.

There are simple methods the patient can use in order to sleep better. We can refer the patient to the information provided in the patient booklet (pg 9) and advise the patient to:

- Keep to regular hours for going to bed and waking up. If the patient sleeps at 10:00 in the night and wake up at 6:00 in the morning, he/she must try to keep to the same timing every day.
- Avoid daytime naps.
- Avoid sleeping tablets or alcohol to fall asleep.
- Avoid tea or coffee after 5 pm.
- Finish any toilet needs just before sleeping.
- If the patient cannot fall asleep easily, he/she must not lie in bed. The patient should get out of bed; try out some activity (e.g. walking, reading a book /religious book or news paper, listen to some pleasant music, etc) and then try to sleep later when feeling really sleepy.
- Have a warm water bath before going to bed in order to relax the body.
- If thinking too much is interfering with sleep, then follow the steps described above. Similarly, feeling fearful or worrying is the reason for poor sleep, practicing the breathing exercise when going to bed will help

DEALING WITH TOBACCO USE

Depression is more common among people who use tobacco (either through smoking or chewing) than in those who don't. One reason may be that those who have experienced depression use tobacco to get relief from a negative mood.

Yet reaching for a cigarette or other tobacco products may not be the solution that it seems to be. Nicotine (a substance contained in tobacco) causes mood swings, helping smokers feel less tense and anxious for a while only to leave them more stressed and depressed between cigarettes. There is some evidence that nicotine can increase the risk of depression in the long run.

There are methods the patient can use in order to stop or reduce use of tobacco. We can refer the patient to the information provided in the patient booklet (pg 10) and advise as below:

If the patient wants to cut down:

- Cut down on one or more cigarettes/bidis/gutka packet than the previous day
- Put off the first use of the day by one or two hours. Go on as long as possible without giving into the craving
- Don't stock up. Buy only the quantity that is planned to be used each day
- On the stop date, stop tobacco altogether and begin life as a non-tobacco user
- If the patient wants to stop at once:
- Set a 'Stop Date'
- Remove all reminders of tobacco use like ashtrays/empty packets etc
- Change the routine

On the 'Stop Date'

- Keep substitutes ready to use like cloves, sweets, etc
- Plan a new routine
- Ask for support from friends and family
- Seek professional support

SUMMARY

- There are some barriers that may prevent effective activation. These may require the use of additional strategies during counselling.
- If repeated negative thoughts or thinking too much are worsening the patient's mood, this can be addressed by solving problems, learning to attend to sensory experience, or using distraction activities
- If the patient has anxiety and reports feeling tense, the breathing exercise may be used to practice relaxation
- If a patient lacks the skills in communicating effectively with others, activation can focus on teaching the patient the skills of communicating. When explaining these strategies to the patient, it us useful to practice them in the session first.
- Patients with Depression often have problems with sleep and tobacco use. Simple suggestions can help them deal with these problems

Chapter 6

Telephone Sessions

Learning Objectives

In this chapter, we will learn:

- How to do telephone sessions to deliver the Healthy Activity Program

CONTENT

The Counselling Relationship manual provides general guidelines for conducting telephone sessions. This section includes additional guidelines that specifically apply to the Healthy Activity Program sessions.

Phase-wise guide for Telephone sessions

Please refer to the complete description for each phase as described in earlier sections of the manual. Special attention is needed in certain areas while delivering sessions on the phone. These areas along with specific guidelines are listed below.

PHASE 1: GETTING STARTED

Patients for whom a face-to-face first session has been completed in the PHC, the telephone counselling guidelines will apply for the next phase.

In the case of patients who have been seen for an abbreviated first session, the full first session may be delivered over the phone.

**Outline**	**Guidelines**
Introducing yourself	Offer a brief introduction as the details would have been shared in the abbreviated first session
Explaining the treatment	Break it down into parts and summarise frequently
Eliciting commitment	Remind the patient of the option of continuing the entire treatment over the phone
Overcoming barriers	Discuss the details of barriers specifically related to continuing the session over the phone
Summarising	Offer frequent summaries and clarify more often throughout all the phases
Completing documentation	Ensure that all documentation is completed at the end of every telephone session

PHASE 2:

Part 1: Learning together

**Outline**	**Guidelines**
Reviewing homework	Encourage the patient to summarise and if the patient finds it difficult, share inputs and ask him/her to summarise in parts
Involving a person close to the patient	You can ask to speak to the person on the phone or you can encourage the person to come and meet you in the clinic when convenient
Planning homework	Ensure that both you and the patient have a copy of the activity plan (pg 23 of the patient booklet) before planning the homework

Part 2: Getting active and solving problems

**Outline**	**Guidelines**
Reviewing homework	As the patient gives feedback on the activity plan, fill in the details in a copy you have so that you can review clearly and maintain this as a record. If the patient is not literate, you may request to talk to the SO who has assisted in completing the chart. Once this is done, get the patient back on the phone and

	summarise what you have understood
Solving problems	Write down the thoughts that are exchanged between the patient and yourself and summarise frequently. If the patient is literate, request them to write down key points like options generated and selected
Planning homework	Request the patient to write down the target activities. If the patient is illiterate, request to speak to a person close to him/her and summarise the target activities along with the instructions

PHASE 3: ENDING WELL

**Outline**	**Guidelines**
Reviewing homework	In case the patient has sought help of a person close to him/her, speak to them as well
Reviewing skills the patient has learnt	Encourage the patient to write down the skills he/she has learnt and if the patient is illiterate, speak to a person close to him/her and summarise the skills

Chapter 7

Role Of Medication

Learning Objectives

In this chapter, we will learn:

- How medicines work in Depression
- When medicines are used in Depression
- Our role when a patient is taking medicines for Depression

CONTENT

The group of medicines that is used in the treatment of Depression is called 'antidepressants'. Antidepressants are an effective alternative or addition to counselling especially in people with severe Depression.

HOW DO MEDICINES WORK IN DEPRESSION?

Though the precise way how antidepressants work is not yet clear, there is enough evidence to suggest that they work by altering the chemical profile of the brain. Antidepressants are carried in the blood to the brain where they attach themselves to particular parts of the nerve cells. Once attached (like a key fitting a lock), these medicines generally increase the available levels of chemicals, which in turn gradually improves the symptoms of Depression.

Understanding how the antidepressants work is important for two reasons.

- Firstly, it takes a few days to two weeks for the patient to start feeling better as the chemical changes in the brain take time to occur. Thus, there is a 'lag period' before the antidepressants begin to show results.
- Secondly, the chemicals that are altered by antidepressants are present in other areas of the body and those also get affected as in the brain. This may lead to side effects like headache, nausea, and diarrhea and other stomach complaints, dry mouth and blurred vision. To put it simply, patients on antidepressants may experience side effects before they start getting better. Both of these possibilities - the delay in starting to feel better and experiencing side effects initially - are important points to communicate to the patient to help support antidepressant treatment.

WHEN ARE MEDICINES USED FOR DEPRESSION?

It is important for us to know the names of the antidepressant so that we recognize them when the doctor prescribes these to a patient we are counselling. The antidepressants most commonly prescribed in the PHC setting are Fluoxetine or Amitryptilline.

Antidepressants may be started along with counselling when the patient is first detected to have Depression if the doctor in the PHC thinks this is necessary.

Antidepressants may be added to ongoing counselling if the patient does not improve after 2 consecutive sessions or worsens despite counselling. We can discuss this with our supervisor who can then speak to the doctor in the PHC.

Some patients may refuse counselling or may not be able to participate in it. In such cases, they can be offered antidepressants as an alternative form of treatment.

WHAT IS OUR ROLE WHEN A PATIENT IS TAKING MEDICINES FOR DEPRESSION?

We can give the patient the following information to help them understand the need to take their medicines regularly as prescribed. While explaining this to the patient, it is useful to refer the patient to pg 12 of the patient booklet:

- Antidepressants are widely used and many people have benefited from antidepressants. They will help in reducing symptoms and improve overall health.
- They are not addictive.
- They may occasionally produce side effects like headache, churning in the stomach, dryness of mouth, etc. These are typically uncommon, mild and short-lived.
- They may take time to begin showing a positive effect on health (up to 2 weeks).

- It is important to take the medicines regularly for maximum benefit.
- It is important to take the medicines as prescribed by one's doctor, even if one is feeling an improvement. If they stop the medicines too soon before the period recommended by the doctor, their problems may return.

Taking medicines regularly is an important activation target and we need to explore barriers that may arise and possible ways to overcome these. For example, if the patient says that he/she may forget to take the medicines, we can discuss ways to remember such as linking taking medicines to meal times, keeping the medicines in a prominent place, involving an SO who can remind the patient when the medicines are due, etc.

If the patient has any unusual side or severe side effects, report these to our supervisor who can then discuss with the doctor

Always encourage the patient to talk with her/his doctor about any questions about medication or side effects.

SUMMARY

- Medicines are as effective as counselling in the treatment of Depression and work by altering the chemical levels in the brain.
- Medicines may be started at the same time as counselling or in patients who do not improve/worsen despite counselling
- When the doctor prescribes medicines for Depression, our role is to continue to provide HAP in the most effective form possible and to support the patient's use of medication by addressing barriers and using activation, as relevant.

APPENDIX 1

Dealing with difficult situations

Problem	Solution
Patient expecting financial assistance	Inform patient that we will work together to identify how financial support can be generated. Emphasise that our role is to offer counselling support
Patient talking too much and not letting the counsellor speak	Set the agenda after introduction and explaining confidentiality in session 1 so that the patient is reassured that she will get a chance to share. If the patient is keen on sharing details first, allow for that. The patient can then be encouraged to return to the agenda with a reminder that further details will be discussed in subsequent sessions.
Patient crying excessively during the session	Allow space for the patient to express her feelings. Do not interrupt or ask too many questions. Provide minimal encouragers and offer a glass of water.
Patient talks about distress/worry related to sexual problems	Be comfortable and objective. Discuss with supervisor and role play in order to learn how to respond to such issues
Patient is grieving over the loss of a loved one	Allow space for the patient to talk. Reflect and help the patient understand that grieving is a natural response to loss. Then encourage the patient to return to the agenda and complete all sessions
Patient not sharing details about the problem, worried about confidentiality	Reassure the patient about privacy
Patient is angry or agitated	Allow the patient to express their feelings. Reflect and suggest that the issues can be discussed in a planned way in the session
Patient is accompanied by small child/children	Suggest that the child/children engage in an activity like drawing that will engage them and allow for a session with fewer interruptions.
Patient is not engaged with the treatment	Focus on the emotions and use skills like reflection. Allow the patient time and space to share thoughts. Encourage the patient to talk about his/her concerns and address these.
Patient does not recover	Refer to the independent psychiatrist who has been contracted for this purpose by the program.

APPENDIX 2

Glossary of difficult words

Difficult words	Meaning/alternate words
Accomplish	Achieve
Acknowledge	To recognize
Agenda	Plan, schedule
Barriers	Difficulties, obstacles
Collaboration	Working together
Commitment	Promise
Communication problem	Difficulty in sharing or saying what one wants to
Distracting	To focus away
Embarrassment	Feel awkward
Encourage	Motivate, support efforts
Handout	Leaflet that provides information
Hyperventilating	Very fast or rapid breathing
Intervention	Provide treatment in order to make a change
Irritated	Annoyed or troubled
Mood ladder	A scale to rate how one is feeling
Phases	Stages or steps
PHQ9	Questionnaire
Postures	The position of the body
Progress	Improvement
Relaxation exercise	Exercise to help one relax, Breathing technique
Review	To look over
Sessions	Sittings, meetings
Solution	To find an answer
Suicide risk assessment	Method to measure the risk of suicide
Summary	Brief of what has been discussed
Supervisors	Experienced seniors
Symptoms	Signs that indicate a disorder
Tingling	Prickly feeling
Trouble shoot	Identify and solve problems
